# POEM: Identifying Joint Additive Effects on Regulatory Circuits

**DOI:** 10.3389/fgene.2016.00048

**Published:** 2016-04-19

**Authors:** Maya Botzman, Aharon Nachshon, Avital Brodt, Irit Gat-Viks

**Affiliations:** Department of Cell Research and Immunology, The George S. Wise Faculty of Life Sciences, Tel Aviv UniversityTel Aviv, Israel

**Keywords:** eQTL, gene modules, pairwise effects, additive effects, immune signaling network

## Abstract

**Motivation:** Expression Quantitative Trait Locus (eQTL) mapping tackles the problem of identifying variation in DNA sequence that have an effect on the transcriptional regulatory network. Major computational efforts are aimed at characterizing the joint effects of several eQTLs acting in concert to govern the expression of the same genes. Yet, progress toward a comprehensive prediction of such joint effects is limited. For example, existing eQTL methods commonly discover interacting loci affecting the expression levels of a module of co-regulated genes. Such “modularization” approaches, however, are focused on epistatic relations and thus have limited utility for the case of additive (non-epistatic) effects.

**Results:** Here we present POEM (Pairwise effect On Expression Modules), a methodology for identifying pairwise eQTL effects on gene modules. POEM is specifically designed to achieve high performance in the case of additive joint effects. We applied POEM to transcription profiles measured in bone marrow-derived dendritic cells across a population of genotyped mice. Our study reveals widespread additive, *trans-*acting pairwise effects on gene modules, characterizes their organizational principles, and highlights high-order interconnections between modules within the immune signaling network. These analyses elucidate the central role of additive pairwise effect in regulatory circuits, and provide computational tools for future investigations into the interplay between eQTLs.

**Availability:** The software described in this article is available at csgi.tau.ac.il/POEM/.

## Introduction

The transcriptional regulatory program that controls the expression of a gene may combine the joint effect of several regulatory mechanisms that act in concert during the cellular response to internal and external signals. These regulatory programs are apparent across a variety of joint contributions, from the independent contribution of each of the regulatory mechanisms to a cooperative contribution of several mechanisms. A regulatory program may include a variety of mechanisms such as transcription factors, chromatin remodeling complexes, and promoter regulatory elements.

Natural genetic variations may provide important insights into regulatory programs. In particular, transcription profiles can be integrated with genotypic data across a population to identify genomic loci that have an effect on gene expression (Mackay et al., 2009), and hence it is possible to use these loci as potential regulatory mechanisms. These mechanisms are referred to as “expression Quantitative Trait Loci” (eQTLs). Multifactorial regulatory programs can be then suggested by finding several eQTLs that are associated with the same gene (e.g., Storey et al., 2005; Huang et al., 2013). The use of natural perturbations, unlike experimental construction of combinations of mutant alleles, is therefore an efficient and non-laborious way to identify regulatory programs in a systematic manner. In this study we focus specifically on regulatory programs with two eQTLs and refer to such programs as “pairwise effects.”

Pairwise effects on gene expression may be classified on the basis of two main categorizations. One major categorization reflects the presence or absence of epistasis. In the absence of epistasis the effect of one eQTL remains the same regardless of the genotype of the other eQTL; the resulting model is therefore said to be “additive” or “non-epistatic.” In the presence of epistasis, by contrast, there is a change in magnitude or direction of one eQTL that depends on the genotype of the other eQTL. “Epistasis” therefore refers to a modification of the additive effects of two loci in a regulatory program (Huang et al., 2013). The other type of categorization reflects the genomic positions of the pairwise effect: none of the loci act at the proximity of the affected gene (termed “*trans*-acting effects”), or alternatively, at least one of the loci acts in the proximity of the gene (termed “*cis*-acting effects”).

Recent eQTL studies systematically analyzed pairwise effects on gene expression. Existing methods have been applied to each gene independently (Brem et al., 2005; Evans et al., 2006; Brown et al., 2014) or on gene groups rather than just a single gene (referred to as “modularization methods”; Kendziorski et al., 2006; Lee et al., 2006; Litvin et al., 2009; Zhang et al., 2010; Kreimer et al., 2012). In particular, current modularization-based eQTL methods are mainly focused on epistasis, tacitly assuming that the effect of one locus is dependent on the particular alleles in the other locus. For example, Kreimer et al. (2012) searched for allele-specific epistasis and Zhang et al. (2010) searched for epistasis with weak marginal effects. They are thus of limited utility for the case of additive joint effects. While several approaches do indeed allow identification of additive relations (Phillips, 2008; Mackay et al., 2009; Huang et al., 2013), these methods do not exploit the modularization in expression and therefore lack the power to detect *trans*-acting loci whose effects are typically weak. Thus, the existing eQTL methods are limited in their ability to provide a comprehensive view of *trans*-acting and additive pairwise eQTL effects.

In accordance, recent studies have shown that pairwise effects mostly involve *cis*-acting rather than *trans*-acting pairs of eQTLs. For example, an investigation of blood from 800 human individuals identified 488 *cis*-*cis*- or *cis*-*trans*-acting pairwise effects but only 13 *trans*-*trans*-acting effects (Hemani et al., 2014). Similarly, analysis of lymphoblastoid cell lines from the TwinsUK cohort yielded 57 pairwise eQTL effects, none of which was a *trans*-*trans*-acting pair (Brown et al., 2014). Many studies have highlighted the key role of additive pairwise effects (Storey et al., 2005; Hill et al., 2008; Bloom et al., 2013), but in most of these reports *trans*-acting eQTL pairs were not mentioned. The apparent scarcity of additive *trans*-acting pairwise effects on gene expression may be due to their low abundance, or a non-comprehensive mapping of additive relations, or both.

Here we describe POEM (Pairwise effect On Expression Modules), a novel methodology that we developed on the basis of iterative refinements of a residual-based stepwise regression (RBSR) model. In our scheme, a “poeModule” is a group of genes (under particular stimulations) that are affected by the same pair of eQTLs. The aim of POEM is to identify poeModules together with their underlying pairwise effects. In particular, it is specifically designed for the case of additive relations. Our analysis of synthetic data demonstrated the superiority of POEM over existing eQTL methods. We applied POEM to a dataset of murine bone-marrow-derived dendritic cells, and found that additive *trans*-acting effects are common (24 poeModules covering 9.8% of the dataset under study), suggesting that the apparent scarcity of such effects in the abovementioned publications (e.g., Storey et al., 2005; Brown et al., 2014; Hemani et al., 2014) is mainly due to their non-comprehensive mapping. Interestingly, the reconstructed model is organized in several multi-poeModule structures of pairwise effects. These multi-poeModule structures offer insights into the capacity of combinatorial regulation of the inflammatory and antiviral signaling pathways in dendritic cells. Together, these results suggest a high prevalence of non-epistatic, *trans*-acting pairwise eQTL effects, and provide a general method for future investigations.

## Materials and methods

### Background and definitions

#### A standard 1-locus scan

We consider a previously measured genotyping of *n* individuals at *m* genetic variants, as well as the levels of *l* traits across the same individuals. Let **Y** = [**y**_1_**y**_2_…**y**_*l*_] be a *n* × *l* matrix of trait measurements, where each column vector **y**_*j*_ represents the measurements of a trait *j* across all *n* individuals. In addition, let **G** = [**g**_1_**g**_2_…**g**_*m*_] be a *n* × *m* matrix of measured genotypes where **g**_*x*_ is a column vector of genotyping of variant *x* across all individuals. For simplicity, we assume a haploid organism or homozygous recombinant inbred strains; therefore, genotypic values for the variants are either −1 or 1. To assess the association between a given trait *j* and a genetic variant *x*, we test the significance of the genetic fixed effect β_*x*_ under the following model:

(1) $$\begin{array}{l} {\text{y}}_{j}=\mu_{x}+{\text{g}}_{x}\beta_{x}+\text{e}, \end{array}$$

where μ_*x*_ is the global mean and **e** represents a *n* × 1 vector of normally distributed error terms. The significance is evaluated through a standard ANOVA *F*-test and the resulting *P*-value is referred to as an *association score*. A standard *1-locus scan* calculates the association scores across all genetic variants. Let **A** be the output (*l* × *m*) *association matrix* where **A**_*jx*_ is the association score between trait *j* and variant *x*.

The identification of eQTLs is based on the association scores in **A**. For example, let *v*_*j*_ be the best-scoring locus of trait *j* where *v*_*j*_ = argmax_*x*_ **A**_*jx*_. In case that **A**_*jv*_*j*__ is statistically significant, we refer to *v*_*j*_ as an *eQTL*. The set of significant best eQTL-trait pairs, referred to as an *eQTL map*, is denoted **V** and defined as follows:

(2) $$\text{V}=\{\left(j,v_{j}\right)\text{|}{\text{A}}_{{jv}_{j}}>T_{j}\text{ and }v_{j}=\underset{x}{\text{argmax }}{\text{A}}_{jx}\},$$

where *T*_*j*_ is a significance cutoff. We note that the POEM algorithm does not utilize Equation (2) to construct the output eQTL map. Instead, it utilizes a revised definition of this map, as formulated in Equation (4) below.

Importantly, it is possible to apply a *conditioned 1-locus scan* that depends on a given eQTL map **V**^0^ from a previous scan. Specifically, in this study we focus on a conditioned scan that utilizes the residuals of the eQTLs in **V**^0^ as the dependent variables, while the calculation of **A** and **V** remains unchanged. Thus, we use the following model:

(3) $$\begin{array}{l} {\text{r}}_{j}=\mu_{x}+{\text{g}}_{x}\beta_{x}+\text{e}, \end{array}$$

We note that Equation (3) is the same as Equation (1) but using a *n* × 1 vector of residual **r**_*j*_ instead of the trait measurements **y**_*j*_. For each trait *j*, **r**_*j*_ is calculated as ${\text{r}}_{j}={\text{y}}_{j}-{\hat{\mu }}_{v_{j}^{0}}-{\text{g}}_{v_{j}^{0}}{\hat{\beta }}_{v_{j}^{0}}$ where $v_{j}^{0}$ is the eQTL of trait *j* in map **V**^0^ (that is, $\left(j,v_{j}^{0}\right)\in {\text{V}}^{0}$). If a trait *j* is not associated with any eQTL (that is, for each possible variant *x*, (*j, x*) ∉ **V**^0^), then we set **r**_*j*_ = **y**_*j*_.

#### A standard 2-loci stepwise regression

In order to identify pairwise effects, the regression model from Equation (1) should be extended into a multiple regression model where the values of a trait are predicted based on the genotyping of two variants. However, such approach entails multiple testing of *m*^2^ pairs of variants. *Stepwise regression* is designed to reduce this complexity by applying two sequential 1-locus scans, with the first scan identifying the “primary” best variant and the second scan detecting the “secondary” best variant given the information about the primary one.

A common stepwise regression approach is the “RBSR,” which is equivalent to the “Either-significant” strategy proposed by Evans et al. (2006). For a given trait *j* RBSR first scans for the best (primary) eQTL and then rescans for an additional (secondary) eQTL conditioned on the primary eQTL. Assuming an additive pairwise effect, the second scan is performed on the residuals of the phenotypic measurements when the effect of the primary eQTL is removed.

Formally, a standard RBSR is applied in two steps. Step 1 applies the standard 1-locus scan using the model and definitions in Equations (1) and (2), respectively. The output of this scan is a map of *primary eQTLs*, denoted **V**^*P*^. We further use the notation **A**^*P*^ to denote the output association matrix of this primary scan. Step 2 applies a conditioned 1-locus scan that depends on the map of the primary eQTLs (using Equation 3 where **V**^0^ = **V**^*P*^); the map of *secondary eQTLs*, denoted **V**^*S*^, is then calculated using Equation (2). We use the notation **A**^*S*^ to denote the resulting association matrix of the secondary scan. Based on this procedure, the pair of eQTLs affecting a given trait *j* is $\left(v_{j}^{P},v_{j}^{S}\right)$ where $\left(j,v_{j}^{P}\right)\in {\text{V}}^{P}$ and $\left(j,v_{j}^{S}\right)\in {\text{V}}^{S}$.

We note that the RBSR method is particularly suitable for the POEM algorithm since it relies on additive pairwise effects. An alternative partition-based approach, which is tailored for epistasis, is detailed in Section Synthetic Data Analysis.

#### Construction of co-association groups

An important subroutine of POEM is revealing groups of traits, where each group is controlled by a single (representative) eQTL. The identification of such *co-association groups* (for simplicity, referred to as *groups*), typically relies on the association matrix from a 1-locus scan across all traits (Lund et al., 2003; Breitling et al., 2008; Mackay et al., 2009). Here we used the c++ implementation of InVamod (Gat-Viks et al., 2013), an agglomerative methodology for the identification of co-association groups together with their representative eQTLs. InVamod takes as input a given association matrix **A**, which was generated based on either a conditioned or a non-conditioned 1-locus scan. Based on this input, InVamod first compiles each single trait *j* as an initial group that is associated with its best-scoring variant *v*_*j*_. Next, it agglomerates groups by merging pairs of groups that are associated with nearby variants. After each merging step, InVamod updates a single representative variant for each group in such a way that it best reflects the composition of traits in the newly generated group.

A key step of the InVamod algorithm is the filtration of non-significant groups based on an association-score cutoff. In this study, the chosen cutoff corresponds to an association *P*-value of 0.01. The output grouping solution **c** is a set of *co-association groups* **c** = {*c*_*v*_} where each group *c*_*v*_ is associated with a single *representative eQTL v*. Notably, this allows revising the standard identification of eQTLs (as in Equation 2) according to the representative loci, thus increasing the overall robustness of the learned model. A revised eQTL map **V**_*c*_, based on a grouping solution **c**, is given by the following definition:

(4) $$\begin{array}{l} {\text{V}}_{c}=\left\{\left(j,v\right)|j\in c_{v}\right\}. \end{array}$$

### The POEM algorithm

POEM is a procedure for identifying pairwise effects of two eQTLs on groups of expression traits. An *expression trait* is the transcriptional response of a particular gene under a certain stimulus across all individuals under study. POEM takes as input a collection of expression traits across individuals and the genotyping of the same individuals. The output is a collection of poeModules, where each poeModule is a group of traits that are associated with a specific pair of eQTLs. Similarly to existing eQTL approaches (Kendziorski et al., 2006; Litvin et al., 2009; Zhang et al., 2010; Kreimer et al., 2012), POEM leverages the modularity of the system and gains statistical power by identifying joint effects on the expression of gene groups. Unlike previous methods, POEM searches for additive rather than epistatic joint effects.

**Figure 1:**
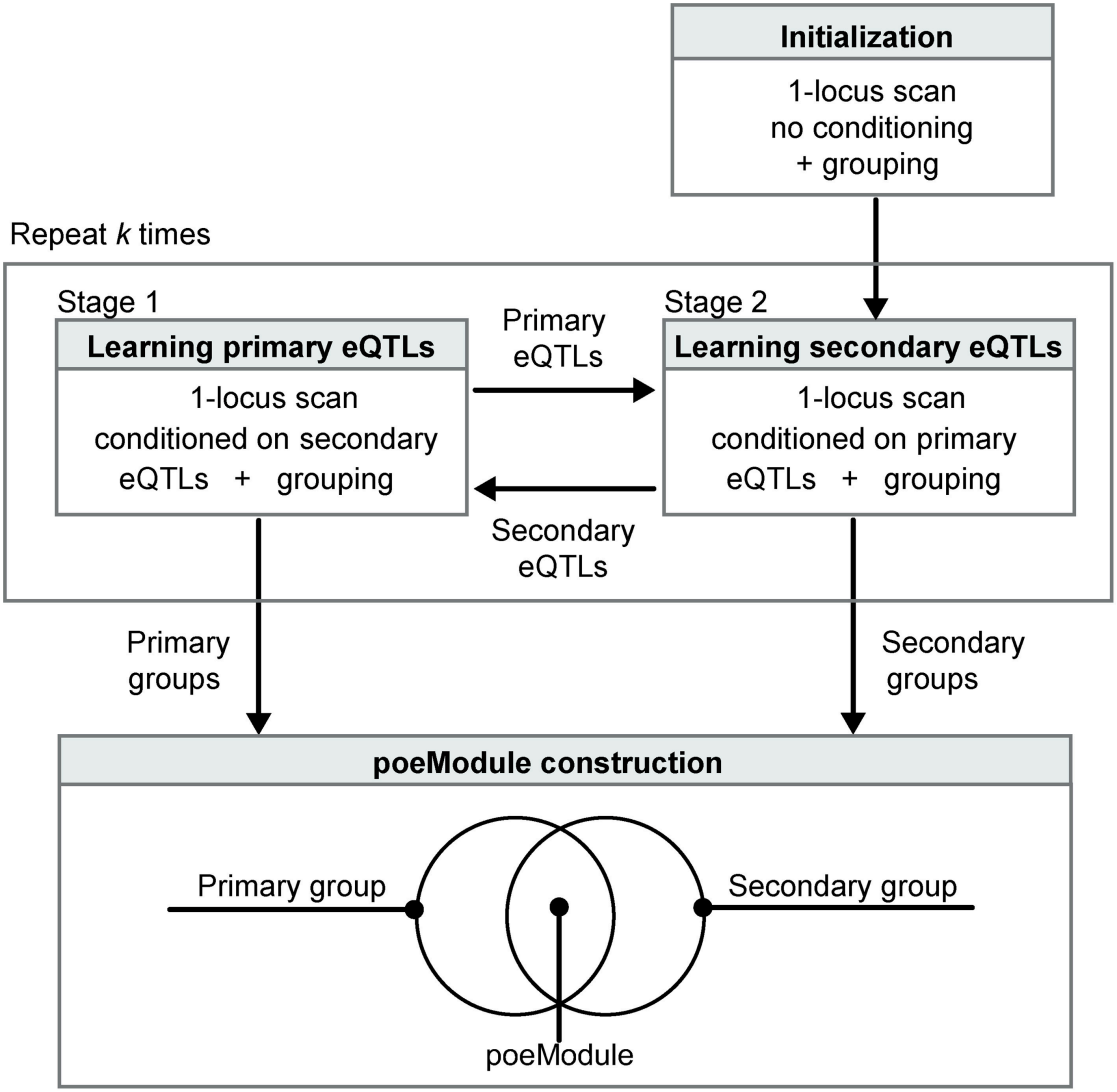
**Overview of the POEM algorithm**. POEM takes as input a collection of expression traits from a certain population of genotyped individuals. The procedure is initiated with a non-conditioned scan **(top right)**. The analysis then consists of two iterative stages: learning primary eQTLs after conditioning on the secondary eQTLs and vice versa **(middle)**. The two steps are repeated *k* times. POEM relies on grouping of the expression traits based on their co-association to the primary and secondary eQTLs. Significant overlaps between the resulting primary and secondary groups are referred to as “poeModules” **(bottom)**. Such poeModules are interpreted as promising pairwise effects that act on the same group of traits.

POEM identifies pairwise effects using the RBSR approach (see above) and further extends RBSR in two ways (Figure 1). First, we expect that grouping of traits can enhance the identification of eQTLs. In accordance, POEM constructs the map of eQTLs on the basis of co-association groups that were generated by the InVamod algorithm. Secondly, a major challenge may arise from an erroneous identification of each eQTL in the presence of the confounding effect of another eQTL, since the pairwise effect may lead to an increased marginal variance. For example, in the RBSR method, the scan for the primary eQTLs is conducted without removing the confounding effect of the secondary eQTLs, and this may blur the primary signals. Consequently, the scan for the secondary eQTLs may also be blurred since it relies on the former inaccuracies in the primary eQTLs. To tackle this challenge, we iterate between two steps. In stage 1—*learning primary eQTLs*—POEM applies a 1-locus scan that is conditioned on the secondary eQTL map, thereby providing a refined collection of primary eQTLs. In stage 2—*learning secondary eQTLs*—POEM applies a 1-locus scan that is conditioned on the primary eQTL map, thus providing a refined collection of secondary eQTLs. Both steps involve grouping of the traits to ensure the robustness of the learned model. We initiate the process with a standard 1-locus scan and then repeat the iterative process *k* times. POEM is implemented in Perl and is publicly available in csgi.tau.ac.il/POEM/. An outline of the POEM algorithm is in Supplementary Figure 1.

#### Stage 1: learning primary eQTLs

In this step, the task is to learn the map of primary eQTLs assuming that the map of secondary eQTLs from stage 2 (denoted **V**${}_{c}^{S}$) is known. POEM first applies a conditioned 1-locus scan where ${\text{V}}^{0}={\text{V}}_{c}^{S}$ (using Equation 3). Let **A**^*P*^ be the output association matrix. Next, InVamod is applied on matrix **A**^*P*^ to generate the co-association groups. As detailed above, this provides (i) a collection of *primary groups*, denoted **c**^*P*^; and (ii) a map of *primary eQTLs*, denoted ${\text{V}}_{c}^{P}$ (generated based on **c**^*P*^ using Equation 4).

#### Stage 2: learning secondary eQTLs

In this step, the task is to learn the secondary groups and secondary eQTLs assuming that the map of primary eQTLs from stage 1 is known. This stage is precisely the procedure we use in stage 1. The only difference is that the input **V**^0^ variable is the map of primary eQTLs. Specifically, we start with a conditioned 1-locus scan where ${\text{V}}^{0}={\text{V}}_{c}^{P}$ (Equations 2 and 3). Let **A**^*S*^ denote the resulting association matrix. We then run the InVamod algorithm on matrix **A**^*S*^ to obtain (i) a collection of *secondary groups*, denoted **c**^*S*^; and (ii) the output map of *secondary eQTLs*, calculated using Equation (4) and denoted ${\text{V}}_{c}^{S}$.

#### Initialization

The initialization stage involves the identification of primary eQTLs in the absence of prior loci. The procedure starts with a (non-conditioned) 1-locus scan (Equation 1), whose output association matrix is then utilized by the InVamod algorithm. As detailed above, the output of the InVamod algorithm includes the initial primary groups, denoted **c**^*P*^, and the initial primary eQTL map (calculated using Equation 4), denoted ${\text{V}}_{c}^{P}$.

#### Construction of poeModules

The final stage of POEM is based on the observation that large overlaps between the primary and secondary groups are unlikely to be generated at random. It is therefore possible to use significant overlaps to infer pairwise effects on groups of traits. In particular, for each pair of a primary group $c_{v_{1}}\in {\text{c}}^{P}$ and a secondary group $c_{v_{2}}\in {\text{c}}^{S}$ , POEM calculates the Fisher's exact test *P*-value for the overrepresentation of overlapping traits *u*_*v*_1_*v*_2__ = *c*_*v*_1__∩*c*_*v*_2__ (assuming that the total number of traits is *l*). We call these *P*-values the *overlap P-values*, and only overlaps with *P*-values that are lower than a certain cutoff are selected. We refer to each significant overlap $u_{v_{1}v_{2}}=\left\{c_{v_{1}}\cap c_{v_{2}}|c_{v_{1}}\in {\text{c}}^{P},c_{v_{2}}\in {\text{c}}^{S}\right\}$ as a *poeModule* and denote the entire collection of poeModules as **U**. Thus, the pair of eQTLs affecting a given trait *j* ∈ *u*_*v*_1_*v*_2__ consists of a primary eQTL *v*_1_ and a secondary eQTL *v*_2_ , where *v*_1_ and *v*_2_ are the representative eQTLs of groups $c_{v_{1}}\in {\text{c}}^{P}$ and $c_{v_{2}}\in {\text{c}}^{S}$ , respectively.

Permutation-based FDR for poeModules were determined by generating 100 permuted gene expression datasets (by random reshuffling of strain labels), running POEM on each of them, and then calculating the ratio between the numbers (or sizes) of the permuted vs. real poeModule.

#### Epistatic vs. additive poeModules

As a final step we aimed to characterize epistatic vs. additive poeModules. We consider a poeModule *u*_*v*_1_*v*_2__ = *c*_*v*_1__ ∩ *c*_*v*_2__ where $c_{v_{1}}\in {\text{c}}^{P}$ denoted the primary group and $c_{v_{2}}\in {\text{c}}^{S}$ denotes the secondary group (with the representative eQTLs *v*_1_ and *v*_2_, respectively). For each trait *j* residing in the poeModule we applied a standard interaction test:

(5) $$\begin{array}{l} {\text{y}}_{j}=\mu +{\text{g}}_{v_{1}}\beta_{v_{1}}+{\text{g}}_{v_{2}}\beta_{v_{2}}+{\text{g}}_{v_{1}}\times {\text{g}}_{v_{2}}\beta_{v_{1}v_{2}}+\text{e}. \end{array}$$

Here, **y**_*j*_ is a *n* × 1 vector of trait measurements; μ is a global mean; **g**_*v*_1__ and **g**_*v*_2__ are *n* × 1 genotyping vectors of eQTLs *v*_1_ and *v*_2_ ; **e** is a *n* × 1 vector of normally distributed error terms; and **g**_*v*_1__ × **g**_*v*_2__ is a *n* × 1 vector of interaction terms, generated by multiplication of the genotypes from each individual. β_*v*_1__ and β_*v*_2__ are the additive effects of the eQTLs and β_*v*_1_*v*_2__ is the interaction term. The *epistasis score* refers to the significance of the interaction term, calculated by testing the alternative hypothesis ${\hat{\beta }}_{v_{1}v_{2}}\neq 0$ against the null hypothesis ${\hat{\beta }}_{v_{1}v_{2}}=0$ (using a standard ANOVA *F*-test). We define an *epistatic poeModule* as a poeModule consisting of at least one epistatically-affected trait (FDR < 0.01). In particular, this was performed by first calculating an epistasis score for the interaction term of each trait, and then correcting for multiple testing using the FDR method. In this study we mainly focus on the non-epistatic poeModules—which are *not* annotated as epistatic ones—referred to as the *additive poeModules*.

### Synthetic data analysis

#### Generation of data

A synthetic collection of expression traits consists of *l* traits that are associated with two eQTLs, denoted *v*_1_ and *v*_2_. The synthetic expression data were generated by means of a two-locus model using Equation (5). Genotypic values for the variants are either −1 or 1, as in the case of homozygous mice. We assume that the additive effect is one-half of the difference in mean trait level between the two genotypic values (with no departure from additivity due to dominance effect). We tested two models: first, an additive model with β_*v*_1__ = β_*v*_2__ = γ , β_*v*_1_*v*_2__ = 0 , and second, an epistasis (co-adaptive) model with β_*v*_1__ = β_*v*_2__ = 0 , β_*v*_1_*v*_2__ = γ , where γ represents the magnitude of effect, referred to as the *genetic effect size*. In the epistasis model, each variant alone has no significant individual effect, but the joint effect of the two variants is notable. In addition, each collection includes *l*_1_ traits that are associated with variant *v*_1_ using the model **y** = μ + **g**_*v*_1__β_*v*_1__ + **e** ; *l*_2_ traits that are associated with *v*_2_ using the model ***y*** = μ + ***g***_*v*_2__β_*v*_2__ + ***e***; and *l*_0_ traits that are not associated with any variant, based on the model **y** = **e**. A single “synthetic collection” consisted of *l* + *l*_1_ + *l*_2_ + *l*_0_ traits and 100 variants, which included the two eQTL variants *v*_1_ and *v*_2_. Each variant was genotyped by randomly sampling the two alleles with equal probabilities.

Overall, we generated *synthetic datasets* of 500 collections, where each dataset was constructed for a certain (additive or epistatic) model, a certain number of individuals (50, 100, or 150), and a given genetic effect size γ (ranging from 0.4 to 1.4). In all cases we used *l* = *l*_1_ = *l*_2_ = 10, *l*_0_ = 50, and σ^2^ = 0.5.

#### Compared methods

We compared the following four alternative methods.

1. *Residual-based stepwise regression (RBSR)*. A stepwise identification of pairwise effects, applied on each trait independently as proposed by Evans et al. (2006). A detailed description of the algorithm appears in Section A Standard 2-Loci Stepwise Regression.
2. *Partition-based stepwise regression (PBSR)*. This method applies a stepwise identification of allele-specific interactions on each trait independently, as described by Brem et al. (2005). For each given trait, the procedure first scans for the best eQTLs (a 1-locus scan that yields the primary eQTL). Identification of the primary eQTL makes it possible to partition the samples into two groups based on the genotyping of the primary eQTL. Next, a separate 1-locus scan is performed for a secondary eQTL in each of the sample groups. Each of the two resulting secondary eQTLs is therefore specific to particular alleles of the primary eQTL.
3. *Partition-based modularization (PBM)*. A standard partition-based modularization method is the “module-network” approach (Segal et al., 2003). However, this approach is not tailored for eQTL data and does not assess module *P*-values. To address this, several advanced methods extend this method for the case of individual variation (Lee et al., 2006; Litvin et al., 2009; Kreimer et al., 2012). Here we applied the most recent approach (Kreimer et al., 2012), which relies on a PBSR model, calculates empirical module *P*-values, and is specifically tailored for the case of transcriptional regulatory programs carrying two eQTLs. The method was applied using the “eSNP_architecture” package implementation (Kreimer et al., 2012). In all cases we used the optimal setting of parameters (i.e., the association *P*-value and module *P*-value cutoff parameters, see Kreimer et al., 2012), which maximized the accuracy scores.
4. Non-iterative *POEM*. Applying POEM with a single iteration (*k* = 1). In this case, the analysis involves two sequential scans: one that does not depend on any additional locus (the initialization stage), and one that is conditioned on the primary loci (POEM's stage 2). For a single trait, this approach is equivalent to RBSR; for multiple traits, POEM further exploits the modularization in the biological system.
5. *POEM*. The full POEM algorithm using multiple iterations (*k* = 6).

By testing the five alternative methods it is possible to assess the utility of a residual-based methods compared to a partition-based methods (PBMs) (methods #1, #4, #5 vs. #2, #3, respectively); to evaluate the advantages of grouping over the analysis of each transcript independently (#4 compared to #1, respectively) and to explore the contribution of the iterative approach (#4 compared to #5).

#### Performance analysis

For determination of the ability of a method to correctly identify pairwise effects on each of the synthetic datasets, all traits in a given dataset are split into two classes: one contains *m*_*p*_ traits associated with both *x*_1_ and *x*_2_, and the other contains the non-associated traits. Based on the predictions of the method, we further split these traits into two additional classes (assuming a certain ANOVA *P*-value cutoff): a “positive” class for a transcript that is predicted to be associated with both *x*_1_ and *x*_2_, and a “negative” class for the remaining traits. For each *P*-value cutoff the true positive, true negative, false positive and false negative counts are then calculated. Next, the area under the receiver operating characteristic (ROC) curve is computed on the basis of the resulting counts, referred to as the *accuracy score*. The accuracy score ranges between 0 for a random solution and 1 for an optimal solution. Since true positive pairwise effects can rarely be detected randomly, an accuracy score of 0.5—which consists of a relatively large amount of true positives—typically reflects a highly informative, non-random solution.

We note that the accuracy score is obtained by testing a range of *P*-value cutoffs, which is the main parameter of interest and serves as input in all compared methods. In all cases, these cutoffs refer to the maximum *P*-value attained by the primary and secondary eQTLs. In the case of RBSR and PBSR, we used the association *P*-values of individual traits. For the case of for PBM we used its module *P*-values (see Kreimer et al., 2012, for details), and for POEM (either using *k* = 1 or *k* = 6) we used the InVamod's *P*-value cutoff. To assess the difference in accuracy between two different methods we compared the corresponding ROC curves using a paired *t*-test (comparing specificity for the same sensitivity levels).

### Mouse data analysis

We investigated the genotyped recombinant inbred BXD mouse strains that were generated by crossing the parental C57BL/6J and DBA/2J strains (Peirce et al., 2004). Gene expression dataset of bone marrow-derived dendritic cell across 43 BXD strains was compiled from a Supplementary Table in a previous publication (Gat-Viks et al., 2013). RNA levels were measured in 403 genes at steady state and 6 h after *in-vitro* stimulation with one of three pathogenic components: lipopolysaccharide (LPS), polyinosinic polycytidylic acid (poly I:C), or Pam3CSK4 (PAM) (in all cases, 6- to 8-week-old females were used). A gene's response to a given stimulation in a given strain was calculated as the difference between the log-transformed expression levels at 6 h after stimulation and at steady state. Overall, the data consisted of 1209 distinct response traits (403 genes × 3 stimulations), each of which is measured across the BXD strains and is referred to as an “*expression trait*.” Genotyping data of 3796 SNPs was downloaded from WebQTL (Wang et al., 2003). POEM was applied with *k* = 6 iterations and an InVamod association-score cutoff corresponding to *P-*value of 0.01. We ran the analysis using an overlap *P* < 10^−6^ for poeModules with two traits and *P* < 10^−3^ for poeModules with three or more traits. Using these parameters, POEM runs for about 2 min to analyze the entire mouse dataset, consisting of 1209 traits across 43 strains.

We define a *cis-acting eQTL* as a locus that is physically located at the proximity of the target gene (< 10 Mbp). A *trans-acting poeModule* (*cis-acting poeModule*) is defined as a poeModule whose fraction of *trans*-*trans*-affected traits is above (below) 0.66. The TLR signaling model for poeModules M14-M18 was generated by adding all target genes whose promoters were bound to the transcription factors Nfkb1,2, Irf3,7, or Stat1,2 (1.5 standard deviations or more) in LPS-stimulated dendritic cells during at least one time point (data from Garber et al., 2012).

## Results

### Synthetic data analysis

To characterize the ability of POEM to reveal pairwise effects, we examined synthetic data of both additive and epistatic (co-adaptive) relations among the underlying eQTLs. In particular, we used a single synthetic collection comprised of two eQTLs and 80 expression traits, where each of these eQTLs affected 20 traits (10 of them affected by both eQTLs) and additional 50 traits were not associated with any of these eQTLs. Overall, a single dataset consisted of 500 collections with three important parameters: the joint-effect model of the two eQTLs (either additive or epistatic effects); the number of individuals; and the genetic effect size. These synthetic datasets and performance evaluations based on an “accuracy score” are described in more detail in the Materials and Methods Section.

We investigated the performance of POEM compared to four alternative methods: a RBSR applied to each transcript independently (the “RBSR” approach; Evans et al., 2006); a partition-based stepwise regression applied to each transcript independently (the “PBSR” approach; Brem et al., 2005); a partition-based modularization approach for a simultaneous grouping of expression traits and identification of their underlying pairwise effects (“PBM”; Kreimer et al., 2012); and POEM without iterations (*k* = 1, “Non-iterative POEM”; see Section Materials and Methods). The comparison provided valuable insights regarding three key components of the POEM algorithm: (i) the residual-based methodology; (ii) the grouping of traits; and (iii) the iterative learning approach. For each POEM component, we first consider the relevant comparison and then discuss the contribution of the particular component to the overall performance.

**Figure 2:**
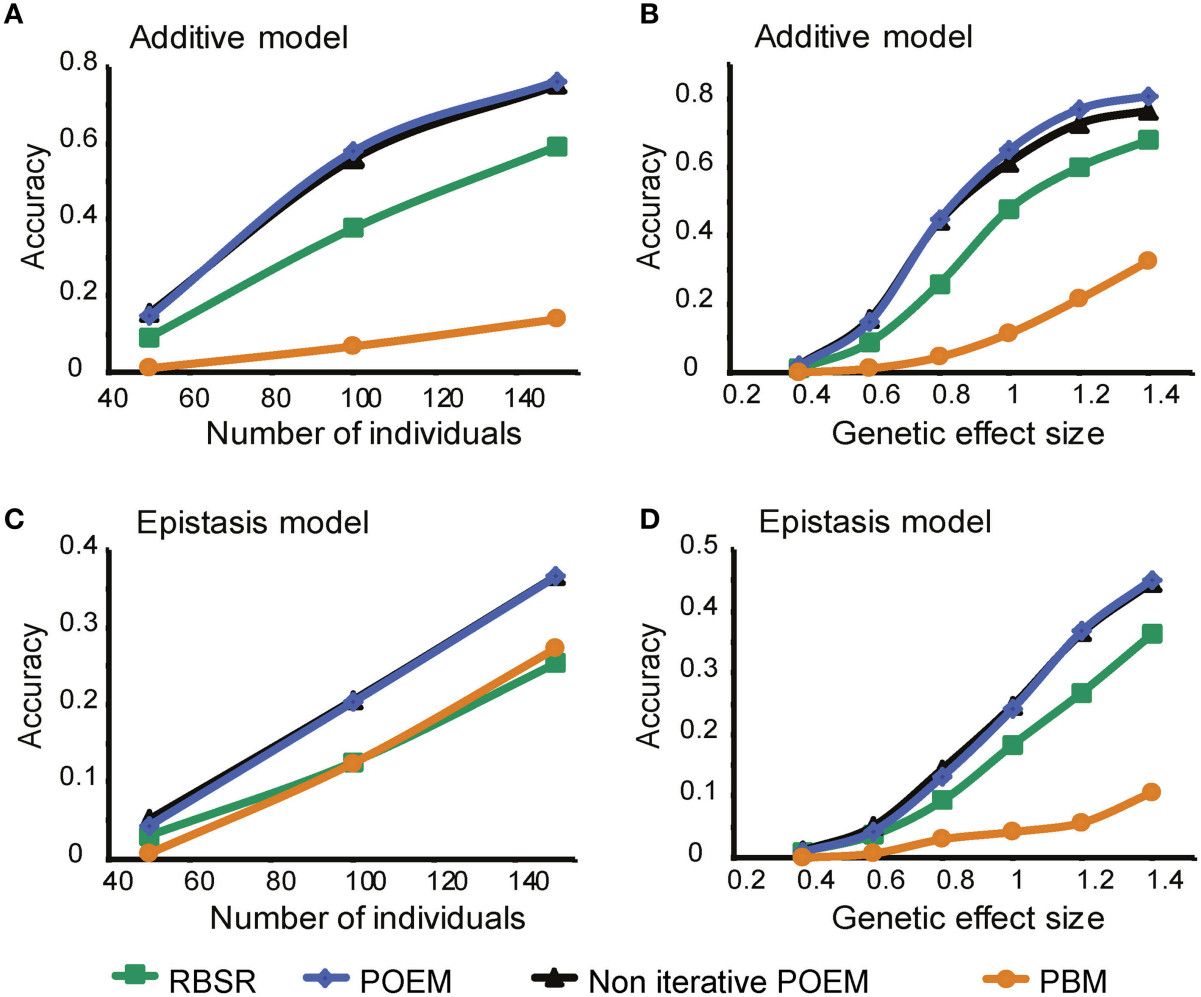
**Performance analysis of the POEM algorithm using synthetic data**. Shown is the accuracy score (*y*-axis) over synthetic datasets that were generated using an additive **(A,B)** or epistasis (co-adaptive; **C**,**D**) model with different numbers of individuals (**A,C**; effect size = 0.6) or different effect sizes (**B,D**, 50 individuals; *x*-axis). The plots demonstrate the improved performance of POEM compared to the alternative methods.

- *The residual-based approach is supported by the simulated data*. We first focused on individual genes and examined which stepwise regression method, either a residual-based (RBSR) or a partition-based (PBSR) approach, performs better in detecting additive effects. We found that the residual-based approach performed well in predicting additive effects (Supplementary Figure 2). These results were qualitatively similar when we used synthetic datasets of varying genetic effect sizes and numbers of individuals (Supplementary Figures 2A,B, *P* < 0.006, 0.039, respectively; paired *t*-test). To confirm the validity of our synthetic data analysis we tested the performance of these methods in the case of a dataset with epistatic effects, where the partition-based approach is expected to perform better since it is tailored for allele-specific alterations. We found that in the case of epistasis the PBM indeed outperformed the residual-based methods (Supplementary Figures 2C,D). Thus, in searching for additive effects, the RBSR approach performed better.
  We next extended the comparison to modularization methods (in addition to the single-trait analysis). Specifically, we investigated the performance of three residual-based methods, namely “RBSR,” “POEM,” and “Non-iterative POEM,” compared to the “PBM.” We found that all three residual-based methods outperformed the partition-based approach when applied to an additive model (*P* < 0.0008, 0.0004, 0.0004, respectively; paired *t*-test; Figures 2A,B), but not necessarily in the epistasis model (Figures 2C,D), as expected. Supplementary Figure 3 further indicates that the residual-based methods attain their best performance in the case of additive pairwise effects, unlike the PBM. These results support the use of residual-based mapping in the additive case.
- *The utility of trait grouping is supported by our simulations*. The POEM algorithm divides the traits into groups, assuming a modular organization of the system. To investigate the utility of grouping we compared between two RBSR-based methodolosgies: first, applying RBSR on each trait independently; and secondly, applying the “non-iterative POEM,” which relies on trait grouping in conjugation with an RBSR technique (in the absence of additional iterative steps). Notably, the performance of the non-iterative POEM approach was significantly better than that attained by the RBSR approach [*P* < 0.0002 (additive model) and *P* < 0.001 (epistasis model); paired *t*-test; Figure 2]. For example, using 100 and 150 individuals, the non-iterative POEM achieved accuracy scores of 0.56 and 0.75, compared to 0.38 and 0.59, respectively, achieved by RSBR (Figure 2A).
- *An iterative refinement of the eQTLs contributes to the performance of POEM*. To evaluate the contribution of POEM's iterative approach, we applied POEM using *k* = 6 iterative steps compared to a single iteration (*k* = 1). We found that in the case of additive effects and large effect sizes, accuracy scores were further increased when the iterative approach was used (“POEM” vs. “Non-iterative POEM,” *P* < 0.0032, paired *t*-test; Figure 2B). The same result was obtained when using different numbers of iterations.

Collectively, the results demonstrated the advantages of POEM, particularly in the case of additive effects, and emphasized the contribution of three key components—grouping, iterative refinement and residual-based analysis—to the POEM algorithm.

### *Trans-*acting, additive pairwise effects are common in murine dendritic cells

We applied POEM to an available dataset of dendritic cells from 43 recombinant inbred mouse strains across 1209 expression traits (Section Materials and Methods; data taken from Gat-Viks et al., 2013). Using a single iteration (*k* = 1) we identified 109 primary groups, 54 secondary groups, and 19 poeModules (Supplementary Figure 4A), with a total of 903, 480, and 89 expression traits, respectively (Supplementary Figure 4B). We observed an increase in the numbers of identified groups and of traits within them during the iterative steps. For example, the number of expression traits in the poeModules increased from 89 to 135 after five additional iterations (*k* = 6; Supplementary Figure 4B, right). Similarly, whereas 33 poeModules were identified after six iterations, only 19 (57%) could be identified after the first iteration (Supplementary Figure 4A, right).

**Figure 3:**
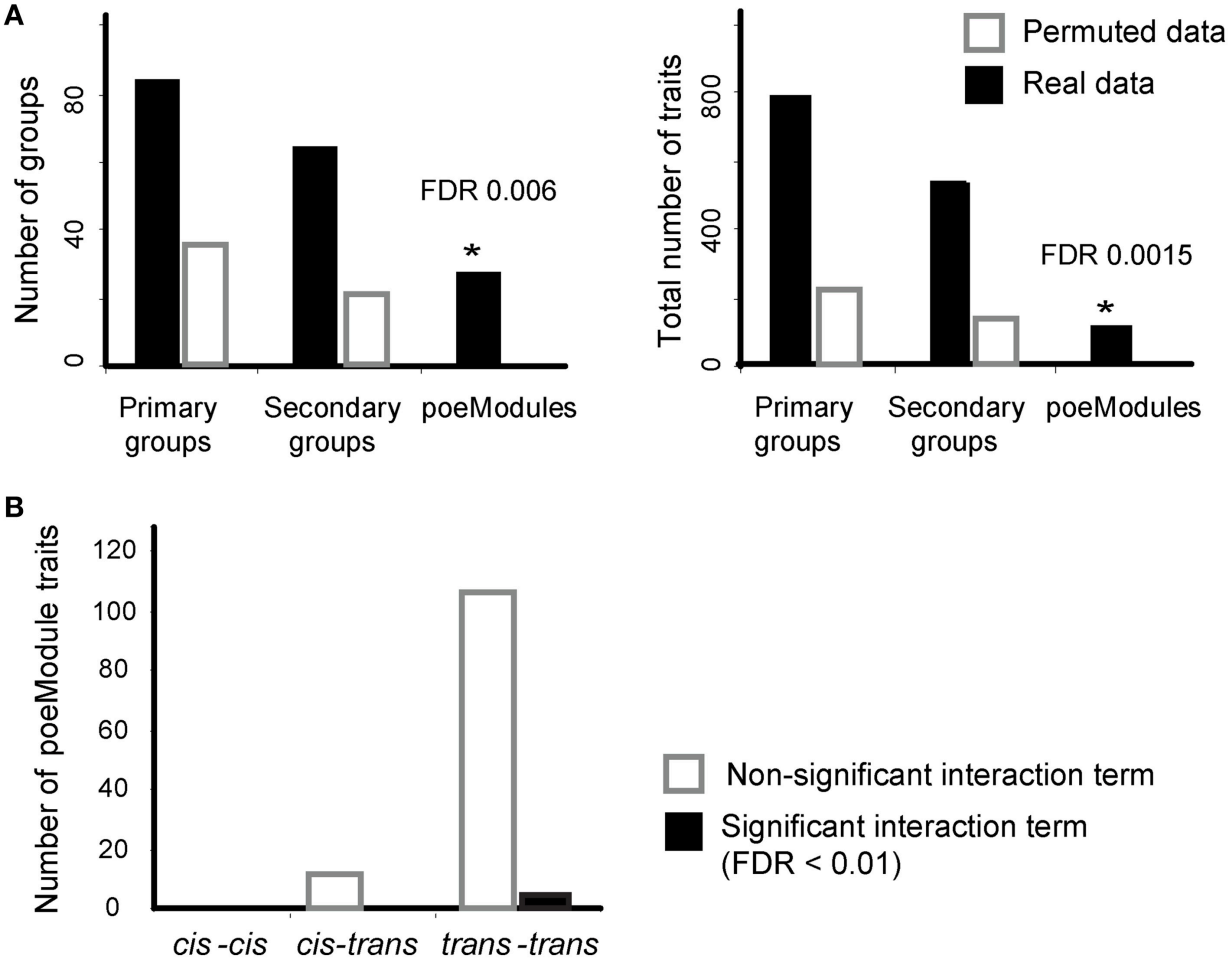
**POEM reveals a widespread *trans-*acting, non-epistatic pairwise effects in murine dendritic cells**. **(A)** Shown are the number of identified groups **(left)** and the number of expression traits within them (**right**, *y*-axis) for real (black) and permuted (white) data across different types of groups (*x*-axis). Based on the permutation test, POEM yielded an empirical false discovery rate (FDR) < 0.006 for predicted poeModules and FDR < 0.0015 for the number of expression traits within the predicted poeModule. **(B)** Shown is the number of identified expression traits within the poeModules (*y*-axis), which are associated by *cis*-*cis*-acting **(left)**, *cis*-*trans-*acting **(middle)**, and *trans-trans-*acting **(right)** eQTL pairs. Significant and nonsignificant interaction terms (FDR < 0.01) are marked in black and white, respectively. ^*^Significant difference

Here we focus on the poeModules that were generated after six iterative steps (*k* = 6). To assess the empirical false discovery rate (FDR) generated by POEM, we repeated the analysis 100 times with permuted gene-expression data generated by randomly shuffling the labels of strains (Section Materials and Methods). Using the permuted data, we found an average of 0.2 poeModules, indicating significant poeModules at FDR < 0.006. Similar results were obtained when we counted the numbers of identified traits within the poeModules (FDR < 0.0015; Figure 3A). This is in contrast to the relatively large number of primary and secondary groups generated using permuted data (leading to FDR < 0.28 and 0.26 for primary and secondary groups, respectively; Figure 3A). These results are in agreement with our selection of a permissive InVamod cutoff (*P* < 0.01) for the generation of intermediate groups, while using a stringent overlap cutoff (*P* < 10^−3^–10^−6^) for the generation of the final poeModules (see Section Materials and Methods). Taken together, although the intermediate groups may consist of false discoveries, POEM successfully controls the FDR in its final poeModules output.

**Figure 4:**
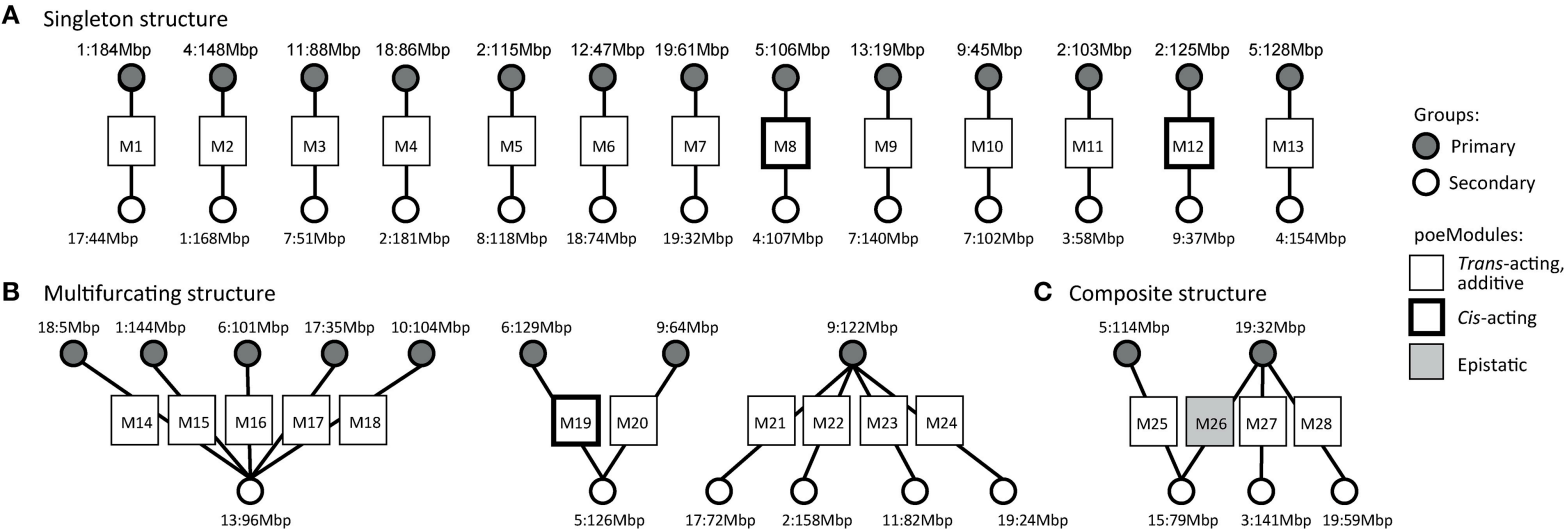
**A high-level organization of pairwise additive effects in murine dendritic cells**. The graph presents poeModules (squares, middle) connected to their primary eQTLs (gray circles, top) and secondary eQTLs (white circles, bottom). On this graph we marked the identifier of the poeModules (M1−M28) and the genomic position peak of each eQTL (see full genomic intervals in Supplementary Table 3). Whereas, most poeModules are *trans*-acting and additive (standard squares), several poeModules are either epistatic (gray squares) or *cis*-acting (bold squares). Notably, whereas some poeModules do not have overlapping primary and secondary eQTLs (“singletons,” **A**), others generate two multi-poeModule structures—the multifurcating **(B)** and composite **(C)** architectures.

We next used only 28 of the 33 resulting poeModules (denoted M1-M28) since the remaining modules were nested within them (Supplementary Tables 1-3). In 25 out of 28 poeModules, at least 66% of the expression traits were affected by two *trans-*acting eQTLs (Supplementary Table 1); such modules are denoted “*trans-*acting poeModules.” In total, 122 of 133 (91%) of the traits in the poeModules were affected by two *trans-*acting eQTLs (Figure 3B). In addition, 27 of the 28 poeModules presented non-epistatic effects: 27 poeModules did not attain significant interaction terms in any of their traits, while module M26 showed significant interactions in four of its traits (FDR < 0.01; Supplementary Tables 1, 2 and Figure 3B; Section Materials and Methods). Overall, POEM mainly identified *trans-*acting, additive poeModules (24 of 28 poeModules; Figure 4), consisting of 118 (9.8%) expression traits that are affected by a pair of *trans-*acting eQTLs carrying a joint additive effect (Supplementary Table 2). These results demonstrate the high prevalence of non-epistatic *trans-*acting pairwise effects and the ability of POEM to reveal them.

### Patterns of interconnections among poeModules

We used our findings in mouse dendritic cells to obtain a global perspective on the organization of poeModules. To this end we constructed a graph of poeModules (Figure 4), on which each poeModule is connected to its primary and secondary eQTLs. Examination of this graph reveals a high-level organization of poeModules. Each poeModule reflects a regulatory program with a unique combination of eQTLs, and some eQTLs are involved in more than one regulatory program. Specifically, we observe 3 characteristic organizations, consisting of “singleton” poeModules and two multi-poeModule structures—the “multifurcating” and “composite” architectures (Figure 4). In the singleton case (13 poeModules, M1−M13; Figure 4A) each eQTL relates to only one poeModule. In the multifurcating structure (Figure 4B), several groups of traits share the same eQTL and have an additional specific eQTL. In particular, we find three multifurcating motifs: one such motif reflects five poeModules (M14-M18) that share the same secondary eQTL in chr13:94–97 Mbp; another bifurcating motif reflects two poeModules (M19 and M20) that share a secondary eQTL in chr5:125–127 Mbp; in the third motif (M21-M24), all traits are affected by a single primary eQTL (chr9:121–124 Mbp), and in addition the traits are divided into four more specific groups in which they have weaker influence from secondary eQTLs (in chr17:69–73 Mbp, chr2:157–160 Mbp, chr11:79–84 Mbp, and chr19:23–26 Mbp). Finally, the composite structure of poeModules M25-M28 (Figure 4C) reflects the possibility that both the primary and the secondary eQTLs may participate in one or a few additional regulatory programs.

**Figure 5:**
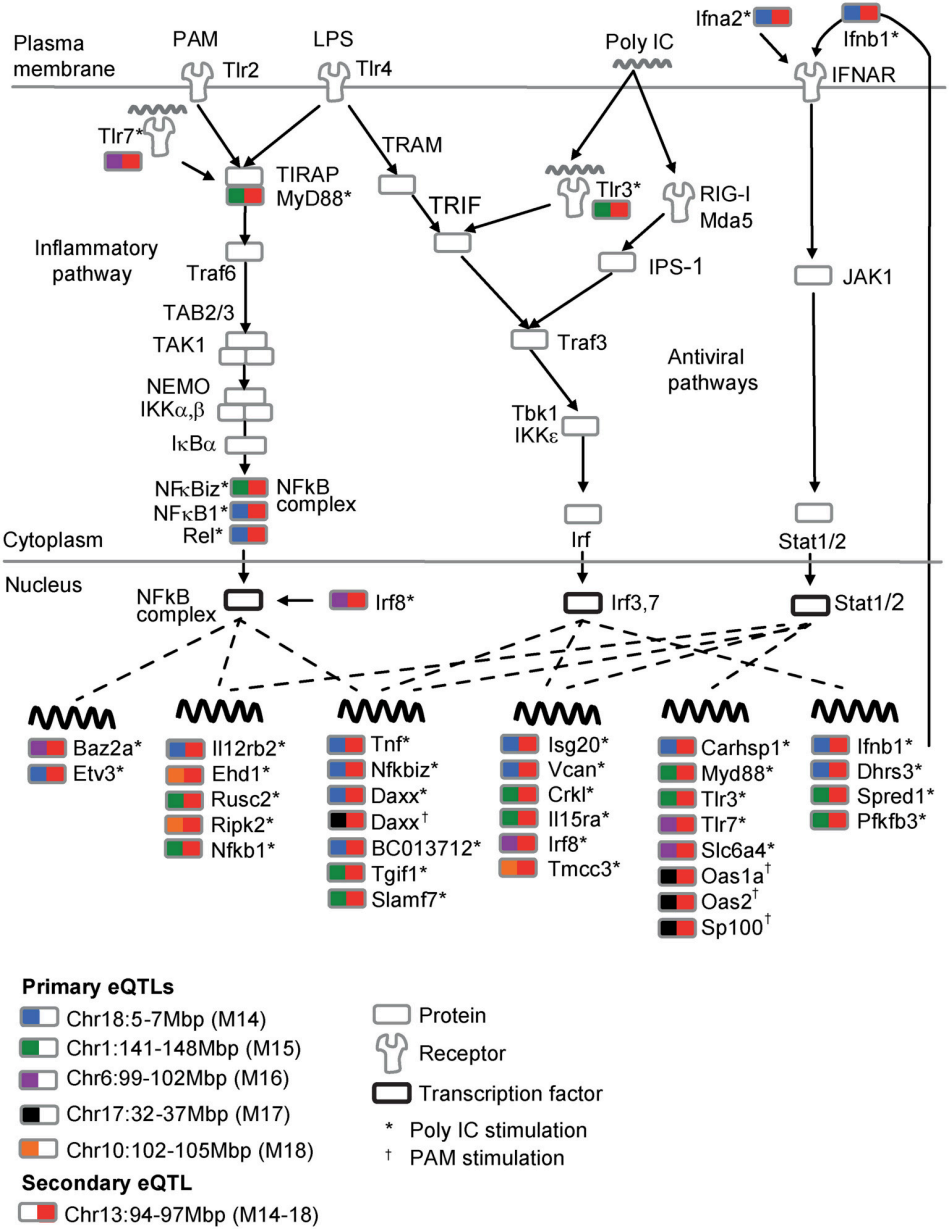
**An integrated model of the TLR signaling pathway modulated by the multifurcating motif of poeModules M14-M18**. Shown is the TLR/RLR signaling pathways in response to the poly I:C, PAM and LPS pathogenic-like ligands. Transcriptional regulation is shown as dashed lines. Each associated trait in poeModules M14-M18 is accompanied with the gene name, the relevant stimulus (^*^poly IC;^†^PAM), and a rectangle that is color coded with its primary (left) and secondary (right) underlying eQTLs. The plot suggests the existence of a single general (secondary) eQTL that acts pleiotropically on the TLR signaling network while cooperating with several specific (primary) eQTLs to control the expression of particular genes within this network.

Next, we demonstrate the multifurcating structure of poeModules M14−M18 (Figure 4B), which relate to five distinct primary eQTLs and share the same secondary eQTL in chr13:94–97 Mbp (Supplementary Table 4). The structure consists of 53 traits that are significantly enriched with Toll-like receptor (TLR) signaling genes (*P* < 0.08, Fisher's exact test). To build a regulatory model of this network, we focused on the particular anti-viral and inflammatory TLR signaling pathways that are triggered by the pathogenic stimulations in our dataset: poly I:C, PAM, and LPS (Figure 5). We included all downstream genes that are directly bound by the key transcription factors in this network (see Section Materials and Methods; Supplementary Table 5). We further annotated each trait (from poeModules M14-M18) in this network with its primary and secondary eQTLs and with its corresponding stimulus. We find that the same signaling pathway is enriched with M14-M18 genes (31 out of 53 genes, *P* < 3 × 10^−6^, Fisher's exact test). Interestingly, whereas a pleiotropic secondary eQTL has an effect on all 31 genes, a variety of primary effects are more specific to particular subsets of genes. For example, three components of the NFκB complex, which plays a key role in TLR signaling, have the same secondary eQTL with distinct primary eQTLs (*Nf*κ*biz* and *Rel* in M14; *Nf*κ*b1* in M15). This observation highlights the central role of combinatorial regulation in molecular processes, and emphasizes the importance of pairwise additive effects for interrogating regulatory circuits.

### Effect of the grouping approach on performance

In an effort to identify co-regulated modules, most modularization methods are focused on co-expression of traits, tacitly assuming that the shared regulatory mechanisms underlie similar expression patterns [e.g., “module networks” (Segal et al., 2003) and MERLIN (Roy et al., 2013)]. For instance, the PCluster algorithm (Friedman, 2003) is a probabilistic agglomerative clustering approach that was used within the “module networks” algorithm. In fact, when performing the optimization solely based on the expression pattern, many of the resulting groups cannot be associated with any of the available genetic variants. Due to this limitation, co-expression cannot be practically used to reveal co-association. To address this problem, a variety of association-based grouping methods have been proposed (e.g., InVamod and NICE; Gat-Viks et al., 2013; Joo et al., 2014). In the association-based grouping techniques, the optimization is performed in a supervised-like manner in order to detect the particular clusters that share the same associated variant. For instance, the InVamod (Gat-Viks et al., 2013) methodology—used in the POEM algorithm—applies a method closely related to an agglomerative clustering, but estimates the coherence of clusters based on association scores rather than measuring dissimilarity between expression profiles.

Here we asked whether the co-association-based grouping played a key role in identifying the poeModules. To this end we compared the co-association-based InVamod algorithm to the co-expression-based PCluster algorithm. When applied to the dataset of dendritic cells, InVamod created 109 groups. PCluster was then applied using this number of groups as input. In most cases, using InVaMod increased the coherence of the association signal within the groups. For example, the results in Supplementary Figure 5 show that the best (most-significant) median association *P*-values of InVamod were better (lower) than those of the PCluster algorithm, with smaller standard deviation (*P* < 2.5 × 10^−9^, < 3 × 10^−26^, respectively; *t*-test). InVamod produced best (most significant) median association *P*-values that are lower than 0.01 in 109 (100%) of the groups, compared to only 76 (70%) of the PCluster groups. The improved predictions of InVamod are in agreement with the absence of genotyping data as prior to the PCluster algorithm. Thus, substitution of InVamod with a co-expression-based grouping method such as PCluster resulted in reduced performance.

## Discussion

Pairwise eQTL effects provide a model for deciphering regulatory programs that act on gene expression. In this study we presented POEM, a novel algorithm for the characterization of gene modules that are affected additively by pairs of eQTLs. POEM relies on trait grouping based on eQTLs inferred by the RBSR method, and further refines the primary and secondary eQTLs in an iterative manner. The poeModules are manifested as significant overlaps between the primary and secondary groups. Whereas, existing eQTL methods centered on joint epistatic effects (e.g., Zhang et al., 2010; Kreimer et al., 2012) or on single traits in the absence of modularization (Brem et al., 2005; Evans et al., 2006; Brown et al., 2014), our approach was designed for additive effects and leverages the modularity of the system to gain statistical power. Reassuringly, our results in synthetic data demonstrate that POEM significantly outperforms the compared eQTL methods (Figure 2). As expected, POEM achieves high performance in the case of additive pairwise effects with lower performance in the case of epistatic effects (Figure 2 and Supplementary Figure 3).

We attribute the success of POEM to four additional factors. First, the refinement of the solution obtained by a classical (residuals-based) stepwise regression approach through a series of iterative refinement steps (real data: Supplementary Figure 4; synthetic data: “POEM” vs. “Non-iterative POEM,” Figure 2). Second, similarly to previous approaches (Kendziorski et al., 2006; Lee et al., 2006; Litvin et al., 2009; Zhang et al., 2010; Kreimer et al., 2012), the statistical robustness achieved by grouping expression traits allowed us to detect pairwise effects that are difficult to identify using a single-trait analysis (“RBSR” vs. “Non-iterative POEM”; Figure 2). Third, in an effort to identify co-regulated modules, most module-identification methods are focused on co-expression of genes, tacitly assuming that the shared regulatory mechanisms underlie similar expression patterns (e.g., Friedman, 2003; Segal et al., 2003). Our analysis suggests that co-association (rather than co-expression) information is particularly suitable for studying groups of traits sharing the same eQTL effect (Supplementary Figure 5).

Finally, the genetic landscape of the murine transcriptome itself appears to be well suited to the identification of additive *trans-*acting pairwise effects. Unlike previous studies that have mainly identified *cis*-acting or epistatic pairwise effects (Litvin et al., 2009; Zhang et al., 2010; Kreimer et al., 2012; Brown et al., 2014; Hemani et al., 2014), POEM reveals high prevalence of gene modules that are affected by *trans-*acting, non-epistatic pairwise effects (24 poeModules; Figures 3, 4; Supplementary Table 1). In view of these characteristics, we believe that POEM is a valuable technique for the mapping of joint additive effects, and may be used to complement existing techniques for the mapping of epistatic effects.

We found that interconnections among pairwise additive effects are common (Figure 4). In particular, the wiring of connections among poeModules uncovers a high-level organization of multifurcating and composite structures. For example, within the multifurcating structure of poeModules M14−M18, which we further characterized (Figure 5), we found two types of eQTLs acting in concert on the TLR signaling network: a general eQTL (chr13:94–97 Mbp) controls the network pleiotropically, whereas other eQTLs are more specific to particular sets of genes within the network. The poeModules and their high-level organization provide a new regulatory model for anti-viral and inflammatory responses in immune cells.

## Author contributions

MB and IG developed the algorithm, performed the statistical evaluation, and wrote the manuscript. AN and AB contributed to the statistical evaluation.

## Funding

This work was supported by the Israeli Science Foundation (1643/13), the European Research Council (637885), and a fellowship from the Edmond J. Safra Center for Bioinformatics at Tel-Aviv University (MB, AB). Research in the IG. Lab is supported by the Israeli Centers of Research Excellence (I-CORE): Center No. 41/11 and the Broad-ISF program (1168/14). IG is a Faculty Fellow of the Edmond J. Safra Center for Bioinformatics at Tel Aviv University and an Alon Fellow.

### Conflict of interest statement

The authors declare that the research was conducted in the absence of any commercial or financial relationships that could be construed as a potential conflict of interest.

## Supplementary material

Supplementary Figure 1 **Outline of the POEM algorithm**.

Supplementary Figure 2 **Comparative performance analysis using synthetic datasets**. Shown are the accuracy scores (y-axis) over synthetic datasets with different genetic effect sizes (a,c; 50 individuals) or different numbers of individuals (b,d; effect size = 0.6; x-axis). Results are shown over synthetic datasets that were generated using additive pairwise effects (a,b) and epistatic relations (c,d). Plots depict two alternative stepwise regression approaches—residuals-based regression (black; as in Evans et al., 2006) and partition-based stepwise regression (gray; as in Brem et al., 2005)—indicating that the residuals-based approach has an advantage in the case of additive pairwise effects.

Supplementary Figure 3 **Performance evaluation**. Shown is the accuracy score (y-axis) of the POEM (top left), PBM (top right), RBSR (bottom left) and non-iterative POEM (bottom right) methods over synthetic datasets with different numbers of individuals (x-axis), which were generated assuming an additive (red) or epistasis (green) model (effect size = 0.6). The three residual-based approaches (POEM, non-iterative POEM and RBSR) have an advantage in the presence of additive effects, whereas the accuracy of the partition-based method (PBM) is better in the case of epistasis.

Supplementary Figure 4 **Primary groups, secondary groups and poeModules in murine dendritic cells. (a)** Numbers of identified primary groups (left) secondary groups (middle) and poeModules (right, y-axis) for real (black) and permuted (blue) data, across varying numbers of POEM iterations (x-axis). **(b)** Total numbers of traits included in the identified primary groups (left), secondary groups (middle) and poeModules (right, y-axis) for real (black) and permuted (blue) data, across varying numbers of POEM iterations (x-axis). Plots indicate that the numbers of primary and secondary groups and the numbers of traits within them are substantially higher in real data than in permuted data.

Supplementary Figure 5 **Comparison of grouping algorithms**. Distributions of median (left) and standard deviation (right) of association P-values across the traits within each group. The groups were generated using the co-association-based InVamod algorithm (black; Gat-Viks et al., 2013) and co-expression-based PCluster algorithm (white; Friedman, 2003). The plots indicate that traits in the InVamod-derived co-association groups have better coherence than traits in the PCluster-derived groups.

Supplementary Table 1 **The identified poeModules in the murine dendritic cells dataset**. Shown are poeModule identifiers (column 1) and types (column 2), their primary and secondary group identifiers (columns 3 and 4), the numbers of expression traits (column 5), and detailed lists of these traits (a gene symbol and a stimulus; column 6). Column 7 records the overlap P-value of each poeModule, and column 8 indicates the global pattern of relationships among the poeModules as detailed in Figure 4. Column 9 indicates the number of traits that are affected by cis-cis or cis-trans eQTL (left) as well as epistatic effects (right) as detailed in columns 5, 7, and 8 of Supplementary Table 2.

Supplementary Table 2 **Expression traits in poeModules identified in murine dendritic cells**. Shown are expression traits (column 1, a gene symbol; column 2, a stimulus), the poeModule in which they reside (column 3; see column 1 in Supplementary Table 1), their primary cis- or trans-acting eQTLs (columns 4 and 5, respectively) and their secondary cis- or trans-acting eQTLs (columns 6 and 7, respectively; group identifiers are as in column 2 of Supplementary Table 3). Column 8 records the P-values of the interaction components between the primary and secondary eQTLs. *Interaction significance with FDR < 0.01.

Supplementary Table 3 **Primary and secondary groups and their eQTLs in the dataset of murine dendritic cells**. Shown are the group types (primary or secondary group, column 1) and their identifiers and numbers of traits (columns 2 and 3). For each group, the table presents the details of its eQTL: the chromosome (column 4) and its genomic interval (columns 5). Shown are only those groups whose eQTLs are part of at least one poeModule, as detailed in Supplementary Table 1.

Supplementary Table 4 **Association scores in poeModules M14-M18**. Shown is the poeModule (column 1), a trait within this poeModule (column 2-gene name; column 3-stimulation) and the association scores (−log P-value) between this trait and the primary (column 4) and secondary (column 5) eQTLs of its poeModule.

Supplementary Table 5 **The multifurcating pattern of poeModules M14-M18**. Shown are five poeModules that are part of the same multifurcating pattern (column 1, see Figure 4b) together with their primary and secondary group identifiers and eQTLs (column 2; see Supplementary Table 3). For each poeModule, the table records the total number of expression traits (column 3) and the TLR-related traits (columns 4,5), including the names of the TLR signaling genes (column 4), the TLR transcriptional target genes (column 5, based on Garber et al., 2012) and the relevant stimulations for each trait: *Poly IC; ^†^PAM.
