# Supplementary material for: POEM: Identifying Joint Additive Effects on Regulatory Circuits
Source: Front Genet. 2016 Apr 19;7:48. doi: 10.3389/fgene.2016.00048 (PMC4835676; doi:10.3389/fgene.2016.00048)
Supplement: Supplementary Figure 1 — Outline of the POEM algorithm. [file Image1.PDF]

Supp. Figure 1

Input
G // Genotyping matrix
Y // Expression traits matrix

Output
U // A collection of poeModules

Identify significant poeModules
//Iteration 1
$\boldsymbol{A}^P$ = 1_locus_scan($\boldsymbol{Y}$, $\boldsymbol{G}$) // Initialization
($\boldsymbol{c}^P$, $\boldsymbol{V}_c^P$)= InVamod($\boldsymbol{A}^P$) // Initialization
$\boldsymbol{A}^S$ = Conditioned_scan($\boldsymbol{Y}$, $\boldsymbol{G}$, $\boldsymbol{V}_c^P$) // Stage 2
($\boldsymbol{c}^S$, $\boldsymbol{V}_c^S$) = InVamod($\boldsymbol{A}^S$) // Stage 2

//Iterations 2...*k*
Repeat *k*-1 times
$\boldsymbol{A}^P$ = Conditioned_scan($\boldsymbol{Y}$, $\boldsymbol{G}$, $\boldsymbol{V}_c^S$) // Stage 1
($\boldsymbol{c}^P$, $\boldsymbol{V}_c^P$) = InVamod($\boldsymbol{A}^P$) // Stage 1
$\boldsymbol{A}^S$ = Conditioned_scan($\boldsymbol{Y}$, $\boldsymbol{G}$, $\boldsymbol{V}_c^P$) // Stage 2
($\boldsymbol{c}^S$, $\boldsymbol{V}_c^S$) = InVamod($\boldsymbol{A}^S$) // Stage 2

//Identification of poeModules
U = Construction_of_poeModules($\boldsymbol{c}^P$, $\boldsymbol{V}_c^P$, $\boldsymbol{c}^S$, $\boldsymbol{V}_{\boldsymbol{c}}^S$)
Return *U*
