# Supplementary figures and images for: POEM: Identifying Joint Additive Effects on Regulatory Circuits
Source: Front Genet. 2016 Apr 19;7:48. doi: 10.3389/fgene.2016.00048 (PMC4835676; doi:10.3389/fgene.2016.00048)

## Supp. Figure 2

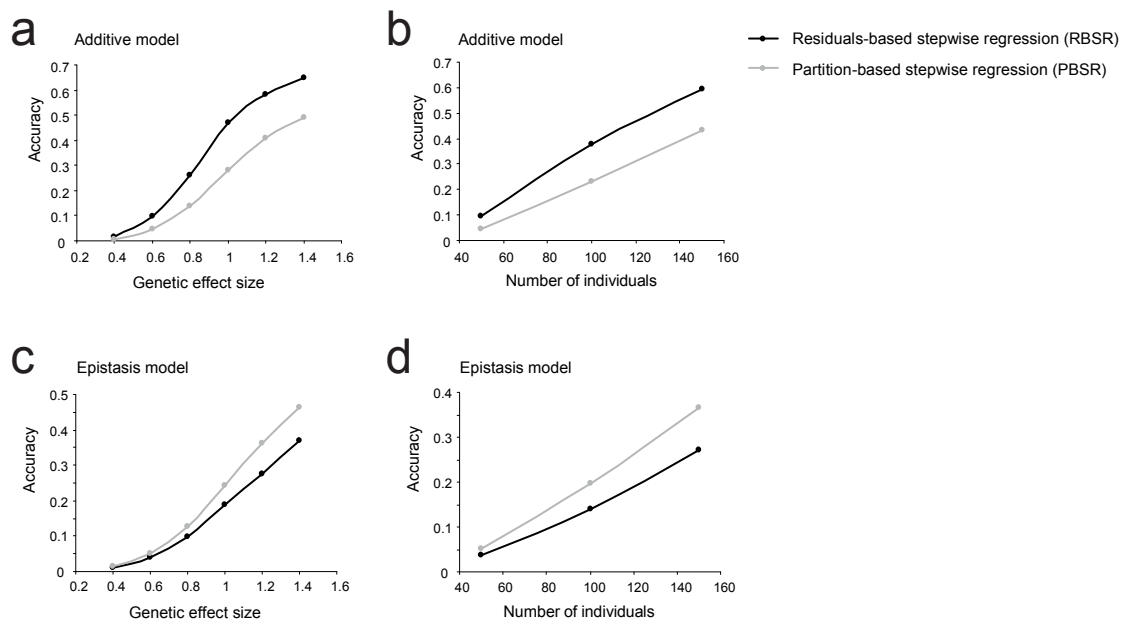

Supplement: Supplementary Figure 2 — Comparative performance analysis using synthetic datasets. Shown are the accuracy scores (y-axis) over synthetic datasets with different genetic effect sizes (a,c; 50 individuals) or different numbers of individuals (b,d; effect size = 0.6; x-axis). Results are shown over synthetic datasets that were generated using additive pairwise effects (a,b) and epistatic relations (c,d). Plots depict two alternative stepwise regression approaches—residuals-based regression (black; as in Evans et al., 2006) and partition-based stepwise regression (gray; as in Brem et al., 2005)—indicating that the residuals-based approach has an advantage in the case of additive pairwise effects. [file Image2.PDF]

Supp. Figure 3

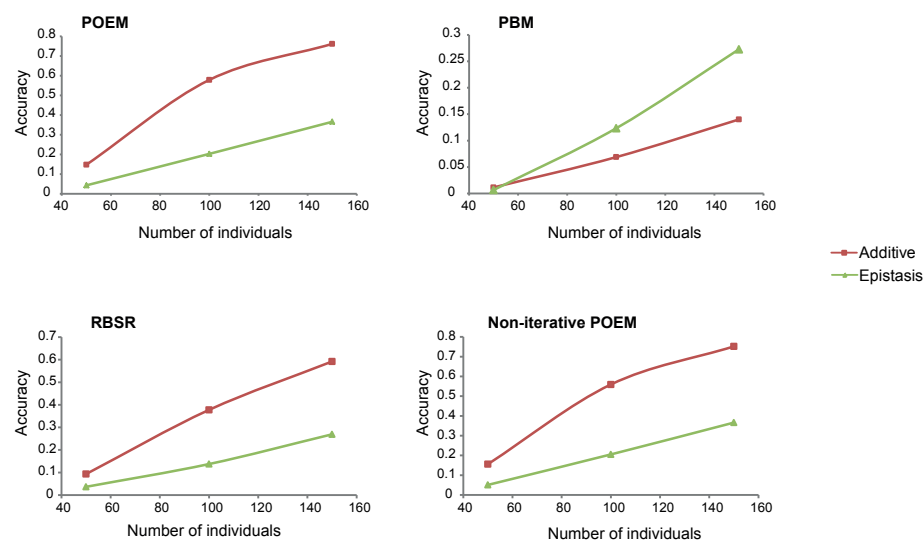

Supplement: Supplementary Figure 3 — Performance evaluation. Shown is the accuracy score (y-axis) of the POEM (top left), PBM (top right), RBSR (bottom left) and non-iterative POEM (bottom right) methods over synthetic datasets with different numbers of individuals (x-axis), which were generated assuming an additive (red) or epistasis (green) model (effect size = 0.6). The three residual-based approaches (POEM, non-iterative POEM and RBSR) have an advantage in the presence of additive effects, whereas the accuracy of the partition-based method (PBM) is better in the case of epistasis. [file Image3.PDF]

Supp. Figure 4

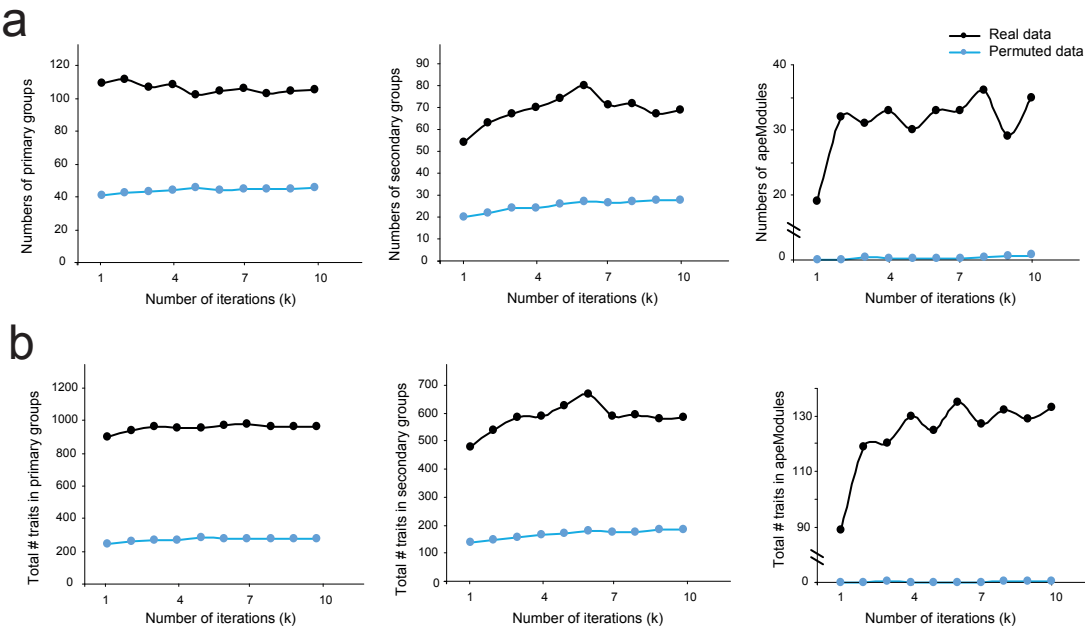

Supplement: Supplementary Figure 4 — Primary groups, secondary groups and poeModules in murine dendritic cells. (a) Numbers of identified primary groups (left) secondary groups (middle) and poeModules (right, y-axis) for real (black) and permuted (blue) data, across varying numbers of POEM iterations (x-axis). (b) Total numbers of traits included in the identified primary groups (left), secondary groups (middle) and poeModules (right, y-axis) for real (black) and permuted (blue) data, across varying numbers of POEM iterations (x-axis). Plots indicate that the numbers of primary and secondary groups and the numbers of traits within them are substantially higher in real data than in permuted data. [file Image4.PDF]

## Supp. Figure 5

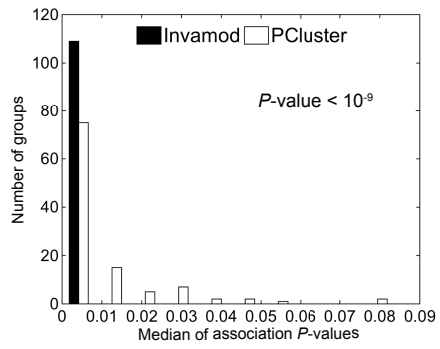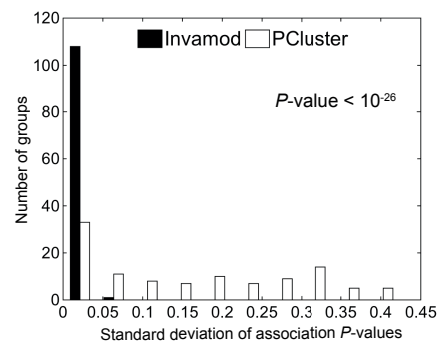

Supplement: Supplementary Figure 5 — Comparison of grouping algorithms. Distributions of median (left) and standard deviation (right) of association P-values across the traits within each group. The groups were generated using the co-association-based InVamod algorithm (black; Gat-Viks et al., 2013) and co-expression-based PCluster algorithm (white; Friedman, 2003). The plots indicate that traits in the InVamod-derived co-association groups have better coherence than traits in the PCluster-derived groups. [file Image5.PDF]
