# Supplementary material for: POEM: Identifying Joint Additive Effects on Regulatory Circuits
Source: Front Genet. 2016 Apr 19;7:48. doi: 10.3389/fgene.2016.00048 (PMC4835676; doi:10.3389/fgene.2016.00048)
Supplement: Supplementary Table 1 — The identified poeModules in the murine dendritic cells dataset. Shown are poeModule identifiers (column 1) and types (column 2), their primary and secondary group identifiers (columns 3 and 4), the numbers of expression traits (column 5), and detailed lists of these traits (a gene symbol and a stimulus; column 6). Column 7 records the overlap P-value of each poeModule, and column 8 indicates the global pattern of relationships among the poeModules as detailed in Figure 4. Column 9 indicates the number of traits that are affected by cis-cis or cis-trans eQTL (left) as well as epistatic effects (right) as detailed in columns 5, 7, and 8 of Supplementary Table 2. [file Table1.PDF]

Supp. Table 1

Module ID	Module type	Primary group	Secondary group	# traits	Traits	Overlap *P* value	Pattern of inter-connections	# cis-affected & epistatically affected traits
M1	*Trans*-acting, additive	P105	S1338	12	Ifit3(poly I:C), Trim21(poly I:C), Dhx58(poly I:C), Ifit2(poly I:C), Cox18(poly I:C), Ifi44(poly I:C), Sp100(poly I:C), Ddx60(poly I:C), Irf7(poly I:C), Oasl2(poly I:C), Iigp1(poly I:C), Enah(poly I:C)	1.2E-28	Singleton	1, 0
M2	*Trans*-acting, additive	P417	S97	4	Tgif1(PAM), Upf3b(LPS), Tgif1(LPS), Pde8a(LPS)	2.3E-16	Singleton	0, 0
M3	*Trans*-acting, additive	P958	S626	4	Oas2(LPS), Ddx60(LPS), Sp100(LPS), Oas1a(LPS)	2.1E-15	Singleton	0, 0
M4	*Trans*-acting, additive	P1432	S246	4	Ets2(PAM), Nfkb1(PAM), Areg(LPS), Cd97(LPS)	2.3E-10	Singleton	0, 0
M5	*Trans*-acting, additive	P199	S750	3	Jdp2(LPS), Tuba4a(poly I:C), Emilin1(LPS)	8.5E-13	Singleton	0, 0
M6	*Trans*-acting, additive	P1014	S1421	3	Ms4a7(LPS), Pmvk(LPS), Idi1(LPS)	3.2E-05	Singleton	0, 0
M7	*Trans*-acting, additive	P1497	S1465	3	Dusp1(poly I:C), Idi1(PAM), Plod2(PAM)	9.3E-04	Singleton	0, 0
M8	*Cis*-acting, additive	P496	S386	2	Lrrc8c(LPS), Lrrc8c(PAM)	4.4E-11	Singleton	2, 0
M9	*Trans*-acting, additive	P1068	S681	2	Cd93(poly I:C), Dnajc2(poly I:C)	6.3E-08	Singleton	0, 0
M10	*Trans*-acting, additive	P777	S654	2	Slc30a1(PAM), Il12a(PAM)	7.9E-08	Singleton	0, 0
M11	*Trans*-acting, additive	P190	S277	2	Slc25a37(PAM), Tnip1(LPS)	1.6E-07	Singleton	0, 0
M12	*Cis*-acting, additive	P205	S771	2	Slc30a4(LPS), Slc30a4(PAM)	4.1E-07	Singleton	2, 0
M13	*Trans*-acting, additive	P512	S424	2	Emr4(LPS), Gpr137b-ps(LPS)	4.1E-07	Singleton	0, 0
M14	*Trans*-acting, additive	P1379	S1107	22	Carhsp1(poly I:C), Isg20(poly I:C), Dhrs3(poly I:C), Rel(poly I:C), Ftsj3(poly I:C), Lad1(poly I:C), Etv3(poly I:C), Kctd14(poly I:C), Fam105a(poly I:C), Acox1(poly I:C), Ifnb1(poly I:C), Vcan(poly I:C), BC013712(poly I:C), Ptger4(poly I:C), Cxcl9(poly I:C), Daxx(poly I:C), Ccl7(poly I:C), Hmgn3(poly I:C), Il12rb2(poly I:C), Ifna2(poly I:C), Tnf(poly I:C), Nfkbiz(poly I:C)	1.2E-38	Multifurcating	1, 0
M15	*Trans*-acting, additive	P82	S1107	14	Atad3a(poly I:C), Crkl(poly I:C), Il15ra(poly I:C), Nfkb1(poly I:C), Tlr3(poly I:C), Spred1(poly I:C), Tgif1(poly I:C), Myd88(poly I:C), Ets2(poly I:C), Sdcbp2(poly I:C), Pfkfb3(poly I:C), Slamf7(poly I:C), Rusc2(poly I:C), Idi1(poly I:C)	4.6E-14	Multifurcating	0, 0
M16	*Trans*-acting, additive	P577	S1107	9	Nolc1(poly I:C), Exosc5(poly I:C), Baz2a(poly I:C), Tlr7(poly I:C), Prmt3(poly I:C), Irf8(poly I:C), Slc6a4(poly I:C), Pdk1(poly I:C), Bysl(poly I:C)	8.5E-12	Multifurcating	0, 0
M17	*Trans*-acting, additive	P1334	S1107	4	Oas2(PAM), Sp100(PAM), Oas1a(PAM), Daxx(PAM)	3.5E-06	Multifurcating	1, 0
M18	*Trans*-acting, additive	P880	S1107	4	Tmcc3(poly I:C), Ripk2(poly I:C), Ehd1(poly I:C), Tk1(poly I:C)	8.1E-05	Multifurcating	0, 0
M19	*Cis*-acting, additive	P594	S508	2	Klrk1(PAM), Klrk1(LPS)	2.3E-07	Multifurcating	2, 0
M20	*Trans*-acting, additive	P791	S508	3	Akr1b8(LPS), Gyk(PAM), Igf1(PAM)	3.6E-08	Multifurcating	0, 0
M21	*Trans*-acting, additive	P831	S1362_1366	4	Net1(LPS), Net1(PAM), Lfng(PAM), Lfng(LPS)	1.9E-06	Multifurcating	0, 0
M22	*Trans*-acting, additive	P831	S218	4	Spred1(LPS), Carhsp1(PAM), Slamf7(LPS), Rasgrp1(PAM)	6.2E-06	Multifurcating	0, 0
M23	*Trans*-acting, additive	P831	S956	3	Sgk1(PAM), Sgk1(LPS), Emr4(poly I:C)	1.3E-07	Multifurcating	0, 0
M24	*Trans*-acting, additive	P831	S1456	3	Ctnnb1(PAM), Ehd1(LPS), Ehd1(PAM)	1.9E-06	Multifurcating	1, 0
M25	*Trans*-acting, additive	P502	S1214	2	Ccdc86(poly I:C), Spsb4(PAM)	8.5E-08	Composite	0, 0
M26	*Trans*-acting, epistatic	P1465	S1214	4	Tnfsf9(poly I:C), Myd116(poly I:C), Cd70(poly I:C), Relb(poly I:C)	8.7E-06	Composite	0, 4
M27	*Trans*-acting, additive	P1465	S309	3	Zfp36l1(PAM), Socs2(LPS), Socs2(PAM)	1.1E-04	Composite	0, 0
M28	*Trans*-acting, additive	P1465	S1495	7	Irf7(PAM), Cd97(PAM), Stat2(PAM), Rasgrp1(LPS), Oasl2(PAM), Ifit3(PAM), Net1(poly I:C)	6.3E-13	Composite	1, 0
