# Supplementary material for: POEM: Identifying Joint Additive Effects on Regulatory Circuits
Source: Front Genet. 2016 Apr 19;7:48. doi: 10.3389/fgene.2016.00048 (PMC4835676; doi:10.3389/fgene.2016.00048)
Supplement: Supplementary Table 2 — Expression traits in poeModules identified in murine dendritic cells. Shown are expression traits (column 1, a gene symbol; column 2, a stimulus), the poeModule in which they reside (column 3; see column 1 in Supplementary Table 1), their primary cis- or trans-acting eQTLs (columns 4 and 5, respectively) and their secondary cis- or trans-acting eQTLs (columns 6 and 7, respectively; group identifiers are as in column 2 of Supplementary Table 3). Column 8 records the P-values of the interaction components between the primary and secondary eQTLs. *Interaction significance with FDR < 0.01. [file Table2.PDF]

Supp. Table 2

Expression trait		poeModule	Primary eQTL		Secondary eQTL		Interaction significance (*P* value)
**Symbol**	**Stimulus**	**Identifier**	**Identifier**	***cis/trans***	**Identifier**	***cis/trans***	
Relb	PIC	M26	P1465	trans	S1214	trans	6.04E-06 *
Tnfsf9	PIC	M26	P1465	trans	S1214	trans	7.81E-06 *
Cd70	PIC	M26	P1465	trans	S1214	trans	1.03E-05 *
Myd116	PIC	M26	P1465	trans	S1214	trans	1.25E-05 *
Tmcc3	PIC	M18	P880	trans	S1107	trans	0.001114
Ms4a7	LPS	M6	P1014	trans	S1421	trans	0.001645
Cd93	PIC	M9	P1068	trans	S681	trans	0.002136
Lfng	LPS	M21	P831	trans	S1362	trans	0.004489
Lrrc8c	LPS	M8	P496	cis	S386	trans	0.006075
Slc25a37	PAM	M11	P190	trans	S277	trans	0.006261
Ripk2	PIC	M18	P880	trans	S1107	trans	0.009283
Net1	LPS	M21	P831	trans	S1362	trans	0.0113
Lad1	PIC	M14	P1379	trans	S1107	trans	0.0122
Rel	PIC	M14	P1379	trans	S1107	trans	0.01552
Il12rb2	PIC	M14	P1379	trans	S1107	trans	0.01866
Slamf7	LPS	M22	P831	trans	S218	trans	0.02144
Lrrc8c	PAM	M8	P496	cis	S386	trans	0.02379
Rusc2	PIC	M15	P82	trans	S1107	trans	0.03174
Slc30a4	PAM	M12	P205	cis	S771	trans	0.03276
Ifna2	PIC	M14	P1379	trans	S1107	trans	0.03994
Carhsp1	PIC	M14	P1379	trans	S1107	trans	0.04152
Idi1	PIC	M15	P82	trans	S1107	trans	0.04392
Spred1	LPS	M22	P831	trans	S218	trans	0.0549
Etv3	PIC	M14	P1379	trans	S1107	trans	0.05565
Ptger4	PIC	M14	P1379	trans	S1107	trans	0.05879
Slc30a4	LPS	M12	P205	cis	S771	trans	0.06484
Nfkbiz	PIC	M14	P1379	trans	S1107	trans	0.06964
Cxcl9	PIC	M14	P1379	trans	S1107	trans	0.08277
Slamf7	PIC	M15	P82	trans	S1107	trans	0.08831
Enah	PIC	M1	P105	cis	S1338	trans	0.09086
Emr4	PIC	M23	P831	trans	S956	trans	0.09995
Carhsp1	PAM	M22	P831	trans	S218	trans	0.116
Klrk1	LPS	M19	P594	cis	S508	trans	0.1207
Daxx	PIC	M14	P1379	trans	S1107	trans	0.1216
Idi1	PAM	M7	P1497	trans	S1465	trans	0.1241
Tnf	PIC	M14	P1379	trans	S1107	trans	0.126
Tlr7	PIC	M16	P577	trans	S1107	trans	0.1322
Ccdc86	PIC	M25	P502	trans	S1214	trans	0.1336
Tnip1	LPS	M11	P190	trans	S277	trans	0.1401
Kctd14	PIC	M14	P1379	trans	S1107	trans	0.1422
Nolc1	PIC	M16	P577	trans	S1107	trans	0.1428
Plod2	PAM	M7	P1497	trans	S1465	trans	0.1437
Fam105a	PIC	M14	P1379	trans	S1107	trans	0.1517
Ets2	PAM	M4	P1432	trans	S246	trans	0.1586
Net1	PAM	M21	P831	trans	S1362	trans	0.1819
Myd88	PIC	M15	P82	trans	S1107	trans	0.1952
Ehd1	PIC	M18	P880	trans	S1107	trans	0.1991
Atad3a	PIC	M15	P82	trans	S1107	trans	0.2149
Rasgrp1	LPS	M28	P1465	trans	S1495	trans	0.2439
Ifnb1	PIC	M14	P1379	trans	S1107	trans	0.2498

Supp. Table 2 - cont.

Expression trait		poeModule	Primary eQTL		Secondary eQTL		Interaction significance (*P* value)
Symbol	Stimulus	Identifier	Identifier	*cis/trans*	Identifier	*cis/trans*	
Sdcbp2	PIC	M15	P82	trans	S1107	trans	0.2569
BC013712	PIC	M14	P1379	trans	S1107	trans	0.266
Ccl7	PIC	M14	P1379	trans	S1107	trans	0.2786
Il15ra	PIC	M15	P82	trans	S1107	trans	0.2831
Gyk	PAM	M20	P791	trans	S508	trans	0.2834
Emilin1	LPS	M5	P199	trans	S750	trans	0.2857
Ctnnb1	PAM	M24	P831	cis	S1456	trans	0.2933
Rasgrp1	PAM	M22	P831	trans	S218	trans	0.2951
Hmgn3	PIC	M14	P1379	trans	S1107	trans	0.2966
Vcan	PIC	M14	P1379	trans	S1107	cis	0.3087
Klrk1	PAM	M19	P594	cis	S508	trans	0.3412
Nfkb1	PIC	M15	P82	trans	S1107	trans	0.349
Sp100	LPS	M3	P958	trans	S626	trans	0.3523
Cd97	LPS	M4	P1432	trans	S246	trans	0.3695
Socs2	LPS	M27	P1465	trans	S309	trans	0.3777
Ftsj3	PIC	M14	P1379	trans	S1107	trans	0.399
Dusp1	PIC	M7	P1497	trans	S1465	trans	0.4111
Tgif1	PAM	M2	P417	trans	S97	trans	0.4115
Oas2	PAM	M17	P1334	trans	S1107	trans	0.4155
Sgk1	LPS	M23	P831	trans	S956	trans	0.4224
Ets2	PIC	M15	P82	trans	S1107	trans	0.423
Isg20	PIC	M14	P1379	trans	S1107	trans	0.4237
Nfkb1	PAM	M4	P1432	trans	S246	trans	0.4433
Slc30a1	PAM	M10	P777	trans	S654	trans	0.4537
Il12a	PAM	M10	P777	trans	S654	trans	0.4591
Baz2a	PIC	M16	P577	trans	S1107	trans	0.4642
Jdp2	LPS	M5	P199	trans	S750	trans	0.4802
Sp100	PAM	M17	P1334	trans	S1107	trans	0.4846
Spsb4	PAM	M25	P502	trans	S1214	trans	0.4897
Igf1	PAM	M20	P791	trans	S508	trans	0.5027
Tk1	PIC	M18	P880	trans	S1107	trans	0.5055
Upf3b	LPS	M2	P417	trans	S97	trans	0.508
Tgif1	PIC	M15	P82	trans	S1107	trans	0.519
Ddx60	LPS	M3	P958	trans	S626	trans	0.521
Spred1	PIC	M15	P82	trans	S1107	trans	0.522
Socs2	PAM	M27	P1465	trans	S309	trans	0.5417
Crkl	PIC	M15	P82	trans	S1107	trans	0.5496
Slc6a4	PIC	M16	P577	trans	S1107	trans	0.5519
Acox1	PIC	M14	P1379	trans	S1107	trans	0.5552
Irf7	PIC	M1	P105	trans	S1338	trans	0.5592
Oas1a	PAM	M17	P1334	trans	S1107	trans	0.5623
Lfng	PAM	M21	P831	trans	S1362	trans	0.5763
Daxx	PAM	M17	P1334	cis	S1107	trans	0.5926
Iigp1	PIC	M1	P105	trans	S1338	trans	0.598
Ehd1	PAM	M24	P831	trans	S1456	trans	0.6005
Ehd1	LPS	M24	P831	trans	S1456	trans	0.6032
Trim21	PIC	M1	P105	trans	S1338	trans	0.6055
Emr4	LPS	M13	P512	trans	S424	trans	0.6164
Gpr137b-ps	LPS	M13	P512	trans	S424	trans	0.624
Areg	LPS	M4	P1432	trans	S246	trans	0.6571

Supp. Table 2 - cont.

Expression trait		poeModule	Primary eQTL		Secondary eQTL		Interaction significance (*P* value)
**Symbol**	**Stimulus**	**Identifier**	**Identifier**	***cis/trans***	**Identifier**	***cis/trans***	
Bysl	PIC	M16	P577	trans	S1107	trans	0.662
Sp100	PIC	M1	P105	trans	S1338	trans	0.678
Stat2	PAM	M28	P1465	trans	S1495	trans	0.6852
Tuba4a	PIC	M5	P199	trans	S750	trans	0.6863
Cox18	PIC	M1	P105	trans	S1338	trans	0.7006
Sgk1	PAM	M23	P831	trans	S956	trans	0.7018
Dnajc2	PIC	M9	P1068	trans	S681	trans	0.7107
Prmt3	PIC	M16	P577	trans	S1107	trans	0.7137
Akr1b8	LPS	M20	P791	trans	S508	trans	0.728
Ddx60	PIC	M1	P105	trans	S1338	trans	0.7658
Pde8a	LPS	M2	P417	trans	S97	trans	0.767
Net1	PIC	M28	P1465	trans	S1495	trans	0.7713
Tgif1	LPS	M2	P417	trans	S97	trans	0.7731
Ifit3	PAM	M28	P1465	cis	S1495	trans	0.7877
Ifit2	PIC	M1	P105	trans	S1338	trans	0.792
Irf7	PAM	M28	P1465	trans	S1495	trans	0.8076
Oasl2	PIC	M1	P105	trans	S1338	trans	0.822
Pfkfb3	PIC	M15	P82	trans	S1107	trans	0.8407
Idi1	LPS	M6	P1014	trans	S1421	trans	0.8472
Dhrs3	PIC	M14	P1379	trans	S1107	trans	0.8483
Ifi44	PIC	M1	P105	trans	S1338	trans	0.8533
Pdk1	PIC	M16	P577	trans	S1107	trans	0.8836
Tlr3	PIC	M15	P82	trans	S1107	trans	0.8898
Zfp36l1	PAM	M27	P1465	trans	S309	trans	0.8938
Oas1a	LPS	M3	P958	trans	S626	trans	0.9104
Oasl2	PAM	M28	P1465	trans	S1495	trans	0.9129
Oas2	LPS	M3	P958	trans	S626	trans	0.9331
Irf8	PIC	M16	P577	trans	S1107	trans	0.9333
Dhx58	PIC	M1	P105	trans	S1338	trans	0.9414
Exosc5	PIC	M16	P577	trans	S1107	trans	0.9448
Cd97	PAM	M28	P1465	trans	S1495	trans	0.9589
Ifit3	PIC	M1	P105	trans	S1338	trans	0.9892
Pmvk	LPS	M6	P1014	trans	S1421	trans	0.9921
