# Supplementary material for: POEM: Identifying Joint Additive Effects on Regulatory Circuits
Source: Front Genet. 2016 Apr 19;7:48. doi: 10.3389/fgene.2016.00048 (PMC4835676; doi:10.3389/fgene.2016.00048)
Supplement: Supplementary Table 3 — Primary and secondary groups and their eQTLs in the dataset of murine dendritic cells. Shown are the group types (primary or secondary group, column 1) and their identifiers and numbers of traits (columns 2 and 3). For each group, the table presents the details of its eQTL: the chromosome (column 4) and its genomic interval (columns 5). Shown are only those groups whose eQTLs are part of at least one poeModule, as detailed in Supplementary Table 1. [file Table3.PDF]

Supp. Table 3

Group			Group's eQTL	
Type	Identifier	Trait count	Chr	Genomic interval (bp)
Primary	P82	50	1	141417668-147391348
Primary	P831	48	9	121825028-123878248
Primary	P1465	47	19	32437861-32747208
Primary	P1379	40	18	5041117-6947944
Primary	P1497	37	19	60875941-61297371
Primary	P105	31	1	183347400-186146231
Primary	P577	25	6	99053858-101797293
Primary	P417	21	4	144118897-149683530
Primary	P1432	19	18	86553541-86749671
Primary	P1014	16	12	47327316-47917726
Primary	P880	16	10	102632507-105018387
Primary	P199	12	2	113667509-115832114
Primary	P777	12	9	41563128-46045437
Primary	P791	12	9	64300970-65312971
Primary	P512	11	5	127383580-128264975
Primary	P1334	11	17	32078991-37015393
Primary	P958	9	11	87177234-88522934
Primary	P190	6	2	102471685-103290657
Primary	P205	6	2	123481211-125704535
Primary	P1068	5	13	18955999-20529561
Primary	P594	4	6	127844938-129560177
Primary	P502	3	5	113588568-115597884
Primary	P496	2	5	104357982-106769482
Secondary	S1107	67	13	94569175-96470955
Secondary	S1421	31	18	73070073-74895830
Secondary	S1338	26	17	43922072-45448429
Secondary	S1465	24	19	32437861-32747208
Secondary	S1214	17	15	78589640-79503135
Secondary	S1495	17	19	58785854-59530841
Secondary	S218	16	2	157295559-159368725
Secondary	S508	15	5	125382920-127039910
Secondary	S246	13	2	180422342-181542229
Secondary	S309	13	3	140100057-142297854
Secondary	S771	11	9	35020042-37454776
Secondary	S626	9	7	47861154-52146495
Secondary	S681	9	7	139448713-142026097
Secondary	S277	9	3	57801765-61072170
Secondary	S1362	7	17	69322298-72598864
Secondary	S1456	7	19	23889912-25045357
Secondary	S386	6	4	104961980-108237103
Secondary	S424	6	4	154079631-154876684
Secondary	S956	5	11	79360701-83521936
Secondary	S97	4	1	164935095-168323143
Secondary	S750	4	8	117676136-118764104
Secondary	S654	4	7	96589291-103132457
