# Supplementary material for: POEM: Identifying Joint Additive Effects on Regulatory Circuits
Source: Front Genet. 2016 Apr 19;7:48. doi: 10.3389/fgene.2016.00048 (PMC4835676; doi:10.3389/fgene.2016.00048)
Supplement: Supplementary Table 4 — Association scores in poeModules M14-M18. Shown is the poeModule (column 1), a trait within this poeModule (column 2-gene name; column 3-stimulation) and the association scores (−log P-value) between this trait and the primary (column 4) and secondary (column 5) eQTLs of its poeModule. [file Table4.PDF]

Supp. Table 4

Module identifier	Expression trait		Association scores	
	Gene name	Stimulus name	Primary eQTL	Secondary eQTL
M14	Carhsp1	poly IC	10.536	9.65269
M14	Tnf	poly IC	11.3442	6.76142
M14	Dhrs3	poly IC	11.6725	6.89347
M14	Vcan	poly IC	13.2194	8.10729
M14	Isg20	poly IC	13.4851	9.48728
M14	Il12rb2	poly IC	13.6073	7.92838
M14	Etv3	poly IC	14.4247	12.0501
M14	Rel	poly IC	14.4787	13.7079
M14	Ifnb1	poly IC	14.813	8.61824
M14	Ifna2	poly IC	16.08	7.62418
M14	Daxx	poly IC	17.2466	9.09308
M14	Nfkbiz	poly IC	18.414	7.55497
M14	BC013712	poly IC	19.2773	12.0164
M15	Pfkfb3	poly IC	8.34026	10.6065
M15	Tlr3	poly IC	11.8914	8.47938
M15	Crkl	poly IC	12.6027	11.4357
M15	Spred1	poly IC	13.1342	7.75321
M15	Slamf7	poly IC	14.738	4.55592
M15	Rusc2	poly IC	14.9179	8.82205
M15	Nfkb1	poly IC	15.5116	11.8512
M15	Il15ra	poly IC	16.5455	9.91566
M15	Tgif1	poly IC	17.3004	8.02997
M15	Myd88	poly IC	17.7704	8.13683
M16	Tlr7	poly IC	9.32118	6.21265
M16	Slc6a4	poly IC	9.78261	6.89171
M16	Baz2a	poly IC	13.0273	8.44049
M16	Irf8	poly IC	14.7915	8.25874
M17	Oas1a	PAM	14.5061	8.02404
M17	Oas2	PAM	14.6713	8.94413
M17	Sp100	PAM	19.3537	9.974
M17	Daxx	PAM	19.5195	11.482
M18	Ripk2	poly IC	15.9329	8.85083
M18	Ehd1	poly IC	15.8301	11.1751
M18	Tmcc3	poly IC	15.0472	7.27542
