# Supplementary material for: POEM: Identifying Joint Additive Effects on Regulatory Circuits
Source: Front Genet. 2016 Apr 19;7:48. doi: 10.3389/fgene.2016.00048 (PMC4835676; doi:10.3389/fgene.2016.00048)
Supplement: Supplementary Table 5 — The multifurcating pattern of poeModules M14-M18. Shown are five poeModules that are part of the same multifurcating pattern (column 1, see Figure 4b) together with their primary and secondary group identifiers and eQTLs (column 2; see Supplementary Table 3). For each poeModule, the table records the total number of expression traits (column 3) and the TLR-related traits (columns 4,5), including the names of the TLR signaling genes (column 4), the TLR transcriptional target genes (column 5, based on Garber et al., 2012) and the relevant stimulations for each trait: *Poly IC; †PAM. [file Table5.PDF]

Supp. Table 5

poeModule Identifier	Primary and secondary groups and eQTLs	Traits count	TLR signaling molecules	Targets of Stat1/2, Irf3/7 and Nfkb1/2
**M14**	P1379 (chr18:5-7Mbp)<br>S1107 (chr13:94-97Mbp)	22	Nfkbiz*, Rel*, Ifnb1*, Tnf*, Ifna2*	Tnf*, Nfkbiz*, Daxx*, Dhrs3*, BC013712*, Etv3*, Il12rb2*, Ifnb1*, Isg20*, Vcan*, Carhsp1*
**M15**	P82 (chr1:141-148Mbp)<br>S1107 (chr13:94-97Mbp)	14	Nfkb1*, Myd88*, Tlr3*	Myd88*, Tlr3*, Nfkb1*, Tgif1*, Crkl*, Spred1*, Il15ra*, Pfkfb3a*, Slamf7*, Rusc2*
**M16**	P577 (chr6:99-102Mbp)<br>S1107 (chr13:94-97Mbp)	9	Irf8*, Tlr7*	Irf8*, Baz2a*, Tlr7*, Slc6a4*
**M17**	P1334 (chr17:32-38Mbp)<br>S1107 (chr13:94-97Mbp)	4	-	Daxx†, Oas1a†, Oas2†, Sp100†
**M18**	P880(chr10:102-106Mbp)<br>S1107 (chr13:94-97Mbp)	4	-	Ehd1*, Tmcc3*, Ripk2*
